# Bias-free solar hydrogen production at 19.8 mA cm^−2^ using perovskite photocathode and lignocellulosic biomass

**DOI:** 10.1038/s41467-022-33435-1

**Published:** 2022-10-03

**Authors:** Yuri Choi, Rashmi Mehrotra, Sang-Hak Lee, Trang Vu Thien Nguyen, Inhui Lee, Jiyeong Kim, Hwa-Young Yang, Hyeonmyeong Oh, Hyunwoo Kim, Jae-Won Lee, Yong Hwan Kim, Sung-Yeon Jang, Ji-Wook Jang, Jungki Ryu

**Affiliations:** 1 Department of Energy Engineering, Ulsan National Institute of Science and Technology (UNIST), Ulsan, 44919 Republic of Korea; 2 School of Energy and Chemical Engineering, UNIST, Ulsan, 44919 Republic of Korea; 3 Department of Wood Science and Engineering, College of Agriculture & Life Sciences, Chonnam National University, Gwangju, 61186 Republic of Korea; 4 Interdisciplinary Program in IT-Bio Convergence System, Chonnam National University, Gwangju, 61186 Republic of Korea; 5 Graduate School of Carbon Neutrality, UNIST, Ulsan, 44919 Republic of Korea; 6 Emergent Hydrogen Technology R&D Center, UNIST, Ulsan, 44919 Republic of Korea

**Keywords:** Solar fuels, Hydrogen fuel, Electrocatalysis, Energy

## Abstract

Solar hydrogen production is one of the ultimate technologies needed to realize a carbon-neutral, sustainable society. However, an energy-intensive water oxidation half-reaction together with the poor performance of conventional inorganic photocatalysts have been big hurdles for practical solar hydrogen production. Here we present a photoelectrochemical cell with a record high photocurrent density of 19.8 mA cm^−2^ for hydrogen production by utilizing a high-performance organic–inorganic halide perovskite as a panchromatic absorber and lignocellulosic biomass as an alternative source of electrons working at lower potentials. In addition, value-added chemicals such as vanillin and acetovanillone are produced via the selective depolymerization of lignin in lignocellulosic biomass while cellulose remains close to intact for further utilization. This study paves the way to improve solar hydrogen productivity and simultaneously realize the effective use of lignocellulosic biomass.

## Abstract (web summary)

While light-driven water splitting offers a means to produce renewable H_2_ fuel, water oxidation limits performances and yields low-value products. Here, authors demonstrate a photoelectrochemical cell that converts lignocellulosic biomass into valuable products alongside H_2_.

## Introduction

Solar hydrogen production is considered the Holy Grail for effectively harnessing unlimited but intermittent solar energy^1–3^. Despite its conceptual simplicity and intensive studies over two decades, it still remains a highly challenging task. Among a series of photo(electro)chemical processes for its realization, water oxidation is the slowest and most difficult process, as it accompanies the transfer of four electrons^4^. Consequently, it requires a high overpotential of >300 mV―the potential of >1.5 V vs. reversible hydrogen electrode (RHE)―at 10 mA cm^−2^ even with expensive state-of-the-art cocatalysts (e.g., Ir- and Ru-based ones)^5,6^. Moreover, oxygen (O_2_) generated from water oxidation causes additional concerns regarding safety, an additional separation process, and high cost^7^.

To avoid such inherent problems, various alternatives to water have been explored as a source of electrons^8^. Biomass can be considered the primary alternative because it is abundant, cheap, clean, and carbon-neutral^9^. Moreover, it can be oxidized at a lower bias with potential economic benefits from its oxidation byproducts^10^. However, critical issues related to global food ethics and economic feasibility remain when using highly processed and expensive chemicals (e.g., glucose, alcohols, etc.) derived from edible biomass^11–14^. In this context, lignocellulosic (LC) biomass can be an ideal and practical alternative. However, LC biomass has been less explored, especially for solar hydrogen production, due to their low solubility and molecular complexity, which result in low conversion yields of LC biomass with poor selectivity.

The low performance of photocatalysts is another critical hurdle for practical solar hydrogen production. Conventional inorganic photocatalysts (e.g., TiO_2_, WO_3_, and BiVO_4_) are cheap and stable but have a relatively large bandgap (2.4~3.2 eV) along with poor optoelectronic properties^15–17^. These intrinsic problems fundamentally limit the effective use of solar flux and often demand external bias, which deteriorates the solar-to-hydrogen (STH) efficiency far below the benchmark for commercialization (~10%)^18–20^. In this regard, lead halide perovskites offer many advantages over conventional photocatalysts: they have a tunable bandgap and energy level, high absorption coefficient, and excellent charge transport^21,22^. Nevertheless, there have been only a few reports of perovskite-based photoelectrodes for solar hydrogen production^23,24^, and no report exists in conjunction with biomass oxidation due to their vulnerability to water.

**Fig. 1:**
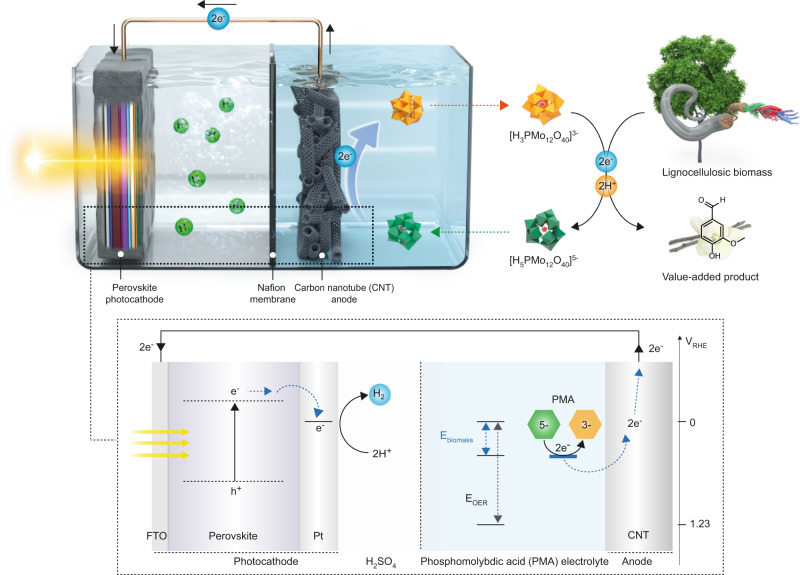
Schematic illustration of biomass–PEC system for unassisted solar hydrogen evolution. Perovskite photocathode with Pt catalyst and electron extraction system from biomass are combined for bias-free hydrogen evolution.

Herein, we report a photoelectrochemical (PEC) cell with a photocurrent density of ~20 mA cm^−2^ by combining a perovskite-based photocathode and LC biomass (Fig. 1). Notably, high solar hydrogen productivity with near-unity faradaic efficiency for hydrogen generation was achieved without any additional bias and the troublesome O_2_ emissions by lowering the potential for an oxidative half-reaction via biomass oxidation (<0.8 V vs. RHE) and harvesting the entire visible light spectrum with a perovskite-based photocathode. Electrons were readily extracted from solid LC biomass by using phosphomolybdic acid (PMA) as a soluble catalyst and an electron/proton mediator. Such configuration can allow the use of biomass in various forms, including insoluble solid biomass such as lignin and lignocellulose that cannot be utilized in direct electrochemical oxidation. Moreover, when using LC biomass, lignin can be selectively depolymerized to produce value-added aromatic compounds via oxidation while other components of lignocellulose such as cellulose and hemicellulose remained close to intact. This solar hydrogen production system stably operated without noticeable performance degradation for 20 h. Our proposed strategy provides insights into the design of advanced solar hydrogen production systems and the broader application of LC biomass for carbon neutrality.

## Results

### Electron extraction and selective depolymerization of LC biomass

To develop biomass-assisted solar hydrogen production systems, we first investigated the extraction of electrons from various biomass. We selected PMA for extraction via biomass depolymerization because it shows a unique color change from yellow (PMA^3−^) to dark green (PMA^5−^) upon reduction―particularly with absorbance at 700 nm, as well as, high solubility and reversible redox behavior^25^ at a relatively low potential (Supplementary Fig. 1). Previous research reported that strong oxidants, such as PMA^3−^ and Fe^3+^, can be reduced while oxidatively depolymerizing biomass and then utilized for electrochemical devices^25–28^. However, these studies have focused on the maximal depolymerization of highly processed biomass-derived chemicals at a relatively high temperature (e.g., by hydrothermal treatment). Even cellulose and hemicellulose, which can be more effectively utilized via biorefinery, were almost completely depolymerized into short-chain aliphatic molecules with a large amount of CO_2_ emissions and lacked a strategic approach for the effective utilization of LC biomass. In this context, we systematically investigated the extraction of electrons from raw LC biomass (from oak) and its macromolecular components (i.e., cellulose, hemicellulose, and lignin)^29^. We hypothesize that lignin in LC biomass can be preferentially depolymerized without altering other biomacromolecules (i.e., cellulose and hemicellulose) and can donate electrons to PMA due to the higher energy and easier oxidation of aromatic groups in lignin than those in cellulose and hemicellulose^30^.

**Fig. 2:**
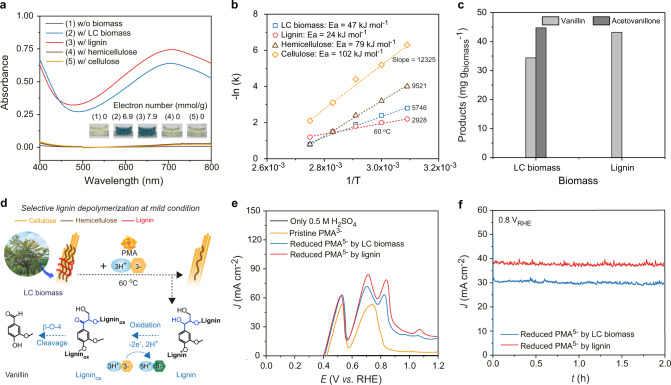
Electron extraction from biomass. **a** UV/Vis spectra of PMA reduction through the oxidation of LC biomass, lignin, hemicellulose, and cellulose at 60 °C for 8 h. **b** Calculated activation energies for PMA reduction by LC biomass, lignin, hemicellulose, and cellulose according to Arrhenius plots. **c** Production of vanillin and acetovanillone from LC biomass, lignin, hemicellulose, and cellulose oxidation. **d** Schematic illustration of electron extraction and production of value-added chemicals from LC biomass via the selective depolymerization. **e** LSV curves of only 0.5 M H_2_SO_4_, pristine PMA^3−^, reduced PMA^5−^ by LC biomass, and reduced PMA^5−^ by lignin. **f** CA curves of reduced PMA^5−^ by LC biomass and lignin at 0.8 V vs. RHE.

The biomasses were incubated at various temperatures of 25, 50, 60, 70, and 90 °C in the presence of PMA (Fig. 2). Interestingly, alkali lignin, and LC biomass can readily be oxidized and donate electrons to PMA in this entire temperature range (practically meaningful above 60°C), whereas, cellulose and hemicellulose can only be oxidized above 90°C (Fig. 2a and Supplementary Fig. 2). According to Arrhenius kinetics, the activation energies for the reduction of PMA by the oxidation of lignin, cellulose, hemicellulose, and LC biomass were 24, 102, 79, and 47 kJ mol^−1^ (0.249, 1.057, 0.819, and 0.487 eV), respectively (Fig. 2b). At 60 °C, the degree of PMA reduction by lignin and LC biomass linearly increased along with incubation time up to 12 h (Supplementary Figs. 3 and 4). After 8 h, PMA extracted 7.9 and 6.9 mmol g^−1^ of electrons from lignin and LC biomass, respectively. These results suggest that lignin is the primary source of electrons even for LC biomass, and that lignin can be depolymerized more preferentially than other macromolecular components of LC biomass (i.e., cellulose and hemicellulose).

To exclude the possibility of complete and non-selective depolymerization of biomass by PMA, we analyzed insoluble and soluble residues of each biomass using various analysis methods before and after the reaction with PMA at 60 °C for 8 h. Fourier-transform infrared spectroscopy showed that the characteristics peaks of lignin between 1400 and 1800 cm^−1^ were weakened after the reaction with PMA, whereas those of cellulose and hemicellulose at ~1100 cm^−1^ were retained (Supplementary Fig. 5). Analysis of LC biomass with 2D ^13^C-^1^H heteronuclear single-quantum correlation nuclear magnetic resonance showed a prominent intensity decrease of the peaks corresponding to lignin after the reaction (Supplementary Fig. 6). Soluble residues of each biomass after the reaction were also analyzed using high-performance liquid chromatography (for cellulose and hemicellulose) and gas chromatography-mass spectrometry (for lignin and LC biomass). Interestingly, 1 g of LC biomass produced 34.1 mg of vanillin and 44.3 mg of acetovanillone, while lignin produced only 43.6 mg of vanillin (Fig. 2c and Supplementary Fig. 7). This additional formation of acetovanillone may be due to the differences between the molecular structure of lignin in LC biomass and lignin obtained by harsh chemical treatments. The formation of these compounds can be attributed to the specific cleavage of the β-O-4 linkage of lignin (Supplementary Fig. 6)^31^. We also confirmed that 50.6% of the original lignin in LC biomass was depolymerized after the oxidation with PMA through the Klason method (Supplementary Fig. 8). Considering that pristine oak contained 23.7% of lignin and 11.7% of lignin remained after the reaction of PMA, ~65% of depolymerized lignin (78.4 mg out of 120 mg) was converted to valuable aromatic compounds such as vanillin and acetovanillone. On the contrary, cellulose and hemicellulose produced negligible amounts of sugars upon reaction with PMA at 60 °C (Supplementary Fig. 9). Notably, no or negligible amounts of CO and CO_2_ were emitted at 60 °C, whereas significant amounts were emitted at 90 °C as reported previously (Supplementary Fig. 10). In addition, scanning electron microscopy showed that the hierarchical porous microstructure of LC biomass was produced after the reaction with PMA due to the selective removal of lignin via depolymerization (Supplementary Fig. 11). These results support our hypothesis that lignin can be selectively depolymerized to generate electrons with the production of value-added compounds such as vanillin and acetovanillone (Fig. 2d). Considering that delignification is the most challenging and energy-intensive process for the biorefinery and pulp industry, these results can provide valuable insights into the utilization of LC biomass in conjunction with electrochemistry. For example, our approach may be utilized for selective fractionation and depolymerization of lignin from lignocellulosic biomass in the future.

The extraction of electrons through the preferential oxidation of lignin in LC biomass can be revealed by considering the microstructure of LC biomass and the two coupled electrochemical half-reactions of biomass oxidation and PMA reduction. Although both cellulose’s glycosidic and lignin’s aryl ether bonds are susceptible to hydrolysis^30,32^, we thought that crystalline nature and burial of cellulose in LC biomass can lead to the preferential depolymerization of lignin in LC biomass. It is well-known that typical LC biomass is composed of microfibrils, in which highly crystalline cellulose is surrounded by amorphous hemicellulose and then lignin. Thus, one would expect lignin to depolymerize more rapidly. On the other hand, the way in which electrons are extracted upon biomass oxidation can be understood in terms of two coupled electrochemical half reactions, as noted in recent studies. Briefly, thermochemical aerobic oxidation reactions (Eq. 1) can be enabled by simultaneous oxidation of substrates (Eq. 2) and reduction of dissolved O_2_ (ORR) (Eq. 3):

1 $$n{{{{{\rm{Su}}}}}}{{{{{{\rm{b}}}}}}}_{{{{{{\rm{red}}}}}}}+{{{{{{\rm{O}}}}}}}_{2}\to n{{{{{\rm{Su}}}}}}{{{{{{\rm{b}}}}}}}_{{{{{{\rm{ox}}}}}}}+{2{{{{{\rm{H}}}}}}}_{2}{{{{{\rm{O}}}}}}$$

2 $$n{{{{{\rm{Su}}}}}}{{{{{{\rm{b}}}}}}}_{{{{{{\rm{red}}}}}}}\to n{{{{{\rm{Su}}}}}}{{{{{{\rm{b}}}}}}}_{{{{{{\rm{ox}}}}}}}+4{{{{{{\rm{H}}}}}}}^{+}+4{e}^{-}$$

3 $${{{{{{\rm{O}}}}}}}_{2}+4{{{{{{\rm{H}}}}}}}^{+}+4{e}^{-}\to 2{{{{{{\rm{H}}}}}}}_{2}{{{{{\rm{O}}}}}}$$

Considering that PMA exhibits facile reversible redox behavior in the potential range similar to that of ORR, one can logically expect that the following reactions (Eqs. 4–6) are also feasible and that the PMA reduction reaction (Eq. 6) will compete with ORR (Eq. 3):

4 $${{{{{\rm{Lignin}}}}}}+{{{{{\rm{PM}}}}}}{{{{{{\rm{A}}}}}}}^{3-}\to {{{{{\rm{Ligni}}}}}}{{{{{{\rm{n}}}}}}}_{{{{{{\rm{ox}}}}}}}+{{{{{\rm{PM}}}}}}{{{{{{\rm{A}}}}}}}^{5-}$$

5 $${{{{{\rm{Lignin}}}}}}\to {{{{{\rm{Ligni}}}}}}{{{{{{\rm{n}}}}}}}_{{{{{{\rm{ox}}}}}}}+2{{{{{{\rm{H}}}}}}}^{+}+2{e}^{-}$$

6 $${{{{{\rm{PM}}}}}}{{{{{{\rm{A}}}}}}}^{3-}+2{{{{{{\rm{H}}}}}}}^{+}+2{e}^{-}\to {{{{{\rm{PM}}}}}}{{{{{{\rm{A}}}}}}}^{5-}$$

Indeed, we found that a continuous supply of O_2_ to the reaction medium significantly lowers the degree of PMA reduction upon reaction with biomass, thus supporting our hypothesis (Supplementary Fig. 12).

The PMAs reduced upon the oxidation of LC biomass were used as an effective source of electrons as an alternative to water. Linear sweep voltammetry (LSV) (Fig. 2e) and chronoamperometry (CA) (Fig. 2f) were conducted using a carbon nanotube (CNT) paper electrode without any catalysts. We found that electrons can be readily extracted through the re-oxidation of the reduced PMAs without generating O_2_ at a potential (<0.8 V vs. RHE) much lower than that of water oxidation even with state-of-the-art electrocatalysts (i.e., >1.5 V vs. RHE). Of note, the efficiency of the PMA re-oxidation was not affected by the presence of oxygen (Supplementary Fig. 13), unlike the PMA reduction by biomass. We observed current densities of ~30 and ~37 mA cm^−2^ at 0.8 V vs. RHE using the PMAs reduced by LC biomass and lignin, respectively (Fig. 2f). In comparison, no current flow was observed when using non-reacted PMAs or raw biomass alone (Supplementary Fig. 14).

### Fabrication of perovskite photocathode for hydrogen production

**Fig. 3:**
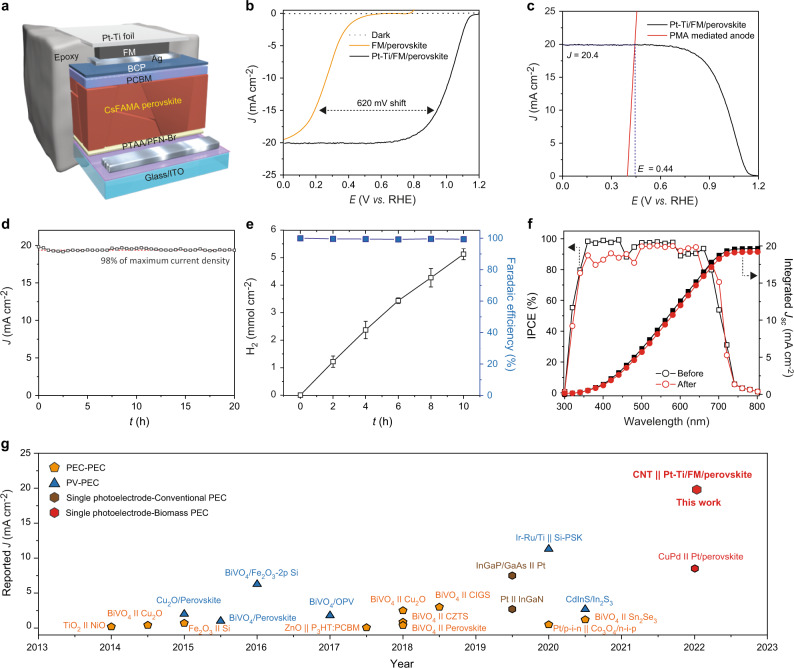
Unassisted PEC hydrogen production. **a** Structure of perovskite layer for photoelectrode. **b** Polarization curves of the perovskite photocathodes with and without Pt catalysts for PEC hydrogen evolution reaction. **c** Estimated theoretical maximum photocurrent density of bias-free PEC cell from the polarization curves of solar hydrogen produced by Pt-Ti/FM/perovskite photocathode and electron extraction from the reduced PMA by the anode. **d** Long-term stability of the bias-free PEC cell. **e** Hydrogen evolution profile of the unassisted PEC system. **f** IPCE spectrum of the bias-free PEC system before and after 20 h stability test. **g** Comparison of the STH efficiency of our device and previously reported solar-to-chemical energy conversion devices. Corresponding references can be found in Supplementary Table 1. All error bars are one statistical s.d.

The low oxidation potential of the reduced PMA encouraged us to attempt a bias-free PEC cell for hydrogen production using a low bandgap photoelectrode that can use solar flux efficiently. We selected organic–inorganic lead halide perovskite for the photoactive material because of its high absorbability from visible to near-infrared spectra, low energy loss, and excellent charge transport stemming from defect tolerance characteristics^33^. Figure 3a shows the detailed architecture of the perovskite-based photocathode. We used Cs_0.05_(FA_0.83_MA_0.17_)_0.95_(PbI_0.83_Br_0.17_)_3_ (CsFAMA) perovskite with a bandgap of 1.61 eV as the light-absorbing layer (Supplementary Fig. 15). In addition, Field’s metal (FM) and Ti foil (0.25 mm) were employed for an excellent electrical connection and high stability under acidic conditions, respectively. The Ti foil was decorated with Pt nanoparticles (20 nm) to facilitate hydrogen reduction (Supplementary Figs. 16 and 17).

The half-cell performance and stability of perovskite-based photocathodes for hydrogen production was measured in 0.5 M H_2_SO_4_ (pH 0.65) under simulated solar irradiation. When the performances of FM-modified perovskite (FM/perovskite), Ti foil-modified FM/perovskite (Ti/FM/perovskite), and Pt particle-deposited Ti/FM/perovskite (Pt-Ti/FM/perovskite) photoelectrodes were compared, the onset potentials for solar hydrogen production were 0.49, 0.62, and 1.14 V vs. RHE, respectively (Fig. 3b and Supplementary Fig. 18). Of note, perovskite photocathodes without any passivation were highly unstable due to their deactivation and dissolution, as reported previously^34^. We found that the passivation of perovskite active layers with both FM and Ti greatly improve the stability of photocathodes. The Ti/FM/perovskite and Pt/Ti/FM/perovskite remained stable for more than 3 h, whereas the un-encapsulated perovskite and the FM/perovskite rapidly lost its performance in a few and 30 min, respectively (Supplementary Figs. 18 and 19).

### Unassisted hydrogen production integrated with biomass system

The biomass-based PEC cell for bias-free solar hydrogen production was fabricated by coupling a perovskite-based photocathode with a CNT paper anode. The perovskite-based photocathode was immersed in the acidic media (0.5 M H_2_SO_4_ in water), while the anode was in 0.5 M H_2_SO_4_ that contained the reduced PMA as an electron mediator. As shown in Fig. 3c, the maximum theoretical photocurrent density of 20.4 mA cm^−2^ (at 0.44 V vs. RHE) was expected from the intersection point of two LSV curves of the photocathode under light (for the hydrogen evolution) and of the anode under dark (for the PMA re-oxidation). Under simulated AM 1.5 G one-sun illumination, the maximum current density (*J*) of 19.8 mA cm^−2^ was obtained with a near-unity Faradaic efficiency without any external bias (Fig. 3d, e and Supplementary Fig. 20 and Supplementary Movie 1). In contrast, no photocurrent was observed when using the unreacted PMA^3−^, confirming the function of the reduced PMA (PMA^5−^) as an alternative source of electrons (Supplementary Fig. 21). The incident photon-to-electron conversion (IPCE) spectrum showed that the PEC cell enabled panchromatic solar hydrogen production (Fig. 3f). Moreover, the PEC device was highly stable under the continuous one-sun illumination for >20 h without considerable performance degradation. Despite the consumption of the reduced PMA (i.e., conversion of PMA^5−^ to PMA^3−^), our PEC device maintained 97% of the maximum photocurrent density. A comparison of cyclic voltammograms before and after the solar hydrogen production showed that PMA remained chemically stable (Supplementary Fig. 22). The observed slight performance degradation can be attributed to the natural properties of perovskite materials and/or the detachment of Pt catalysts. Although there was no change in the optical properties of perovskite active material (Supplementary Fig. 23), it is well-known that the perovskite layer can be degraded due to ion migration and accumulation under continuous illumination^35^. SEM and inductively coupled plasma-mass spectroscopy analyses showed that Pt was partly stripped or corroded after 20 h. Nevertheless, the stability of our PEC device under continuous illumination is still very surprising compared to other studies about perovskite photovoltaics^36^ and photoelectrochemical cells^37,38^.

## Discussion

The achieved bias-free photocurrent density of 19.8 mA m^−2^ corresponds to solar to hydrogen conversion productivity of 512 μmol h^−1^ cm^−2^ and is the record-high value reported to date among solar hydrogen production systems with a single photoelectrode, regardless of device type, such as PEC and photovoltaic-assisted PEC/electrochemical devices for water splitting (Fig. 3g, Supplementary Tables 1 and 2). The STH efficiency of our system can be calculated using the following equation related with the amount of energy consumed to produce hydrogen^39^:

7 $${STH}={\left[\frac{\left|\, {j}_{{SC}}({{{{{\rm{mA}}}}}} \, {{{{{\rm{c}}}}}}{{{{{{\rm{m}}}}}}}^{-2})\right|\times \left({V}_{th}\right)\times {\eta }_{{{{{{\rm{F}}}}}}}}{{P}_{{total}}({{{{{\rm{mW}}}}}} \, {{{{{\rm{c}}}}}}{{{{{{\rm{m}}}}}}}^{-2})}\right]}_{{{{{{\rm{AM}}}}}}1.5{{{{{\rm{G}}}}}}}$$

where *j*_*SC*_ is the short-circuit photocurrent density, and *V*_*th*_ is the thermodynamic electrolysis potential (i.e., 1.23 V for overall water splitting and 0.62 V for our system^27^). Then, the STH efficiency of our systems is 12.3%.

**Fig. 4:**
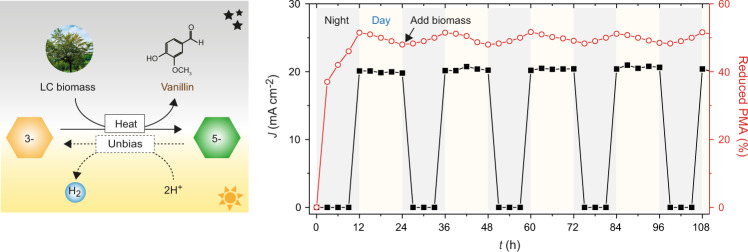
Continuous and repeated operation of biomass PEC systems. PMA was reduced upon concurrent depolymerization and valorization of LC biomass at night (at 60 °C for 12 h), then used to produce hydrogen efficiently during the daytime (under simulated solar irradiation for 12 h).

From a practical viewpoint, our biomass PEC device has many advantages over conventional solar hydrogen production and biomass utilization systems. For example, our biomass PEC system can enable continuous production of valuable chemicals via biomass depolymerization at night and STH conversion during the daytime (Fig. 4), whereas conventional PEC systems can only produce hydrogen during the daytime. Compared to overall solar water-splitting devices, the biomass PEC device allows more effective STH conversion by alleviating the stringent requirement of a high-bandgap photoelectrode and improving the kinetics of oxidation half-reactions. Furthermore, the biomass PEC system neither necessitates the use of expensive catalysts for oxidation half-reactions (e.g., Ru- and Ir-based catalysts) nor generates problematic O_2_. In addition, the PMA-mediated extraction of electrons from biomass can be more useful in practice compared to direct biomass oxidation on the electrode surface: most macromolecular biomasses are insoluble in water, and direct oxidation can depolymerize biomasses non-selectively. However, there can be the physical loss of PMAs upon the repeated cycles of separation and recollection of PMA ex-situ. To address this issue, we are developing an in-situ flow system, which will be reported in due course.

To summarize, we reported a record high photocurrent density of ~20 mA cm^−2^ by coupling solar hydrogen production using a low-bandgap perovskite-based photocathode and PMA-mediated biomass oxidation. Under these optimized conditions, PMA can preferentially depolymerize lignin in LC biomasses and can extract and store electrons, as well as produce value-added chemicals (e.g., vanillin and acetovanillone). The reduced PMA can readily be reoxidized at a relatively low potential and, thus, can effectively replace water as a source of electrons for solar hydrogen production. The reduced energy requirement for solar hydrogen production using biomass allows a low-bandgap light absorber, such as perovskite, to utilize solar flux more effectively and yields record high STH efficiency. Our study paves the way for the realization of solar hydrogen production and effective use of LC biomass for a sustainable future.

## Methods

### Materials

Lignin (alkali type), PMA, nitric acid (≥65%), sulfuric acid (95.0% +), anhydrous N,N-dimethylformamide (DMF), dimethyl sulfoxide (DMSO), chlorobenzene, toluene, methanol, ethyl acetate, isopropanol, oleylamine, and poly(methylmethacrylate) (PMMA) were purchased from Sigma-Aldrich (USA). Oak (>100 mesh) was acquired as LC biomass from Prof. Jae-Won Lee’s group at Chonnam National University. Multi-walled carbon nanotubes (MWCNTs, >95%, OD: 5–15 nm) were purchased from US Research Nanomaterials, Inc. (USA). Ti foil (0.25 μm thick), FM, cesium iodide (CsI), and bathocuproin (BCP, 98%) were purchased from Alfa Aesar (USA). PTAA (poly[bis(4-phenyl)(2,4,6-trimethylphenyl)amine]) and PFN-Br (poly(9,9-bis(3’-(N,N-dimethyl)-N-ethylammonium-propyl-2,7-fluorene)-alt-2,7-(9,9-dioctylfluorene))dibromide) were purchased from Xi’an Polymer Light Technology Corp (China) and 1-Material, respectively. Formamidium iodide (FAI) and methyl-ammonium bromide (MABr) were purchased from Greatcell Solar Materials (Australia). Lead (II) iodide (PbI_2_) and lead (II) bromide (PbBr_2_) were purchased from Tokyo Chemical Industry (TCI, Japan). PC_61_BM was purchased from EM Index.

### Pre-reduction of PMA through biomass oxidation

PMA was dissolved at 0.25 M in 10 mL of 0.5 M H_2_SO_4_. After sonication for 10 min, 0.375 g of lignin, hemicellulose, cellulose, or LC biomass (oak) was added to the PMA solution and then incubated at a constant temperature in an oil bath. Unless stated otherwise, the mixed solutions of PMA and biomass were incubated at 60 °C for 8 h. We calculated the number of electrons extracted from biomass by PMA using the following equation:

8 $${{{{{\rm{moles}}}}}}\; {{{{{\rm{of}}}}}}\; {{{{{\rm{electrons}}}}}}\; {{{{{\rm{per}}}}}}\; {{{{{\rm{g}}}}}}\; {{{{{\rm{lignin}}}}}}=n\times {C}_{{PMA}}\times {V}_{{PMA}}\times {f}_{{red}}/{m}_{{lignin}}$$

where *n* is the number of electrons participating in a redox reaction (2 for reduction of PMA^3−^ to PMA^5−^); *C*_*PMA*_ is the molar concentration of PMA; *V*_*PMA*_ is the volume of the PMA solution; *f*_*red*_ is the fraction of reduced PMA; and *m*_lignin_ is the mass of lignin used for electron extraction. The *f*_*red*_ values were determined by measuring absorbance at 700 nm using the Beer–Lambert law.

The activation energy for biomass oxidation by PMA (*E*_*a*_) was determined using its temperature-dependent kinetics according to the Arrhenius equation:

9 $${{{{{\mathrm{ln}}}}}}(k)=-\!{E}_{a}/RT+\,{{{{{\mathrm{ln}}}}}}(A)$$

where *k* is the rate constant of a certain reaction; *R* is the gas constant; *T* is a reaction temperature; and *A* is the pre-exponential factor.

### Characterization

UV/visible absorbance spectra of PMA solutions were measured using a V-730 UV/vis spectrophotometer (JASCO, Japan). Spectroscopic analyses of lignin, hemicellulose, cellulose, and LC biomass before and after PMA reaction were carried out with a 670/620 Fourier-transform infrared spectrometer (Agilent, USA) in an ATR mode and a VNMRS 600 nuclear magnetic resonance (NMR) spectrometer (Agilent, USA). Samples for GC analysis were collected from the headspace of a sealed vial or H-cell using a gas-tight syringe and were analyzed with a GC-2010 Plus gas chromatograph (Shimadzu Co., Japan). The photovoltaic properties of the perovskite solar cells were recorded using Keithley 2401 source unit under AM 1.5 G (100 mW cm^−2^) illumination by a Class AAA Oriel Sol3A solar simulator (Newport, USA). The spectral mismatch was calibrated using a KG-5 filter-covered monosilicon detector. The optical properties of perovskite solar cells were measured using a V-670 UV/vis spectrometer (JASCO, Japan) and a Varian Cary Eclipse fluorescence spectrometer (Agilent, USA) at an excitation wavelength of 440 nm. The structure of LC biomass, cross-sectional images of perovskite film, and the morphology of platinum nanoparticles on the Ti foil were observed using a NOVA Nano scanning electron microscopy (FEI, USA) equipped with an energy-dispersive X-ray spectrometer. An ESCALAB 250XI UV photoelectron spectrometer (Thermo Fisher Scientific, USA) was used to acquire the UPS spectra of perovskite thin films. Pt nanoparticles on the Ti foil were analyzed using a JEM 2100 transmission electron microscope (JEOL, Japan). The dissolved Pt nanoparticles from Ti-Pt was analyzed using a 700-ES inductively coupled plasma-optical emission spectrometry (Varian, USA).

### Extraction and quantification of vanillin and acetovanillone

Oxidative depolymerization of lignin and LC biomass produced vanillin and acetovanillone. A PMA solution was incubated with lignin or LC biomass at 60 °C, filtered to remove insoluble aggregates, and mixed with chloroform at a 1:1 volume ratio to extract aromatic compounds. Once phase separation occurred, 1 µL of n-decane was added to 2 mL of the chloroform solution as an internal standard for gas chromatography-mass spectrometry (GC-MS) to identify and quantify aromatic compounds, such as vanillin and acetovanillone. GC-MS spectra were measured with a 450-GC gas chromatograph and a 320-MS mass spectrometer (Bruker, USA) equipped with an Rtx-5MS capillary column (30 m × 0.25 mm × 0.25 mm; Restek). Split injections (1 µL) were performed with a GC Pal autosampler (CTC Analytics AG, Switzerland) at a split ratio of 25:1, using He as a carrier gas.

### Fabrication of perovskite solar cells

We washed patterned ITO substrates in ultrasonic baths of acetone and then isopropanol for 20 min each. The pre-cleaned substrates were oven-dried and treated with UV/ozone for 30 min. Subsequently, the ITO substrates were transferred to an N_2_-filled glove box with H_2_O and O_2_ levels below 0.1 ppm. The hole transporting PTAA solution (2 mg mL^−1^ in toluene) was spin-casted at 6000 rpm for 30 s, followed by heating at 100 °C for 15 min. After cooling down to room temperature, PFN-Br solution (0.4 mg mL^−1^ in methanol) was deposited onto the PTAA/ITO films. The triple-cation perovskite precursor solution was prepared by dissolving CsI (0.06 M), FAI (1 M), MABr (0.2 M), PbI_2_ (1.1 M), and PbBr_2_ (0.2 M) in 1 mL of the DMF/DMSO (4/1, v/v) mixed solution. To increase the crystal orientation of the perovskite layer, a trace amount of oleylamine (0.1 wt%) was added to the precursor. A two-step spin-coating procedure at 1000 rpm for 10 s and 5000 rpm for 30 s was adopted to deposit the perovskite layer. During the second step, 200 µL of anti-solvent chlorobenzene was quickly dropped on the center 15 s prior to the end of the spinning process. The obtained light brown film was then annealed at 100 °C for 30 min. For surface passivation treatment, PMMA (0.5 mg mL^−1^ in ethyl acetate) was dropped on top of the films and spin-coated at 5000 rpm, then heated at 100 °C for 5 min. The electron transporting layer (ETL) was deposited by spin-casting the PC_61_BM solution (25 mg mL^−1^ in chlorobenzene) at 1000 rpm for 30 s and BCP in isopropyl alcohol solution (1 mg mL^−1^) at 4000 rpm for 30 s. Finally, a 150-nm-thick Ag electrode was deposited via thermal evaporation at <10^–6^ Torr.

### Fabrication of perovskite photocathodes for solar hydrogen production

Perovskite photocathodes were fabricated based on the perovskite solar cells inside the glove box with O_2_ < 0.1 ppm, and H_2_O < 0.1 ppm and continuous N_2_ supply. The 2.5 cm × 2.5 cm perovskite solar cells were cut into four cells using a diamond cutter knife. Each cell’s active area was then covered with 0.3 cm × 0.3 cm of FM bar and heated at 70 °C for 2 min. The Pt-Ti foil, prepared from the 20 nm of Pt nanoparticles deposited on the Ti foil by e-beam evaporation, was attached to the underlying solar cell once the FM was melted. The edges were encapsulated with epoxy (J-B Weld, USA), and a copper wire was attached to the counter electrode of the perovskite solar cell. The prepared perovskite photocathodes were left for 12 h at room temperature to dry the epoxy sealant.

### Electrochemical characterization

LSV and CA were conducted using an SP-150 Biologic potentiostat (BioLogic Science Instruments, France). The oxidation of the prereduced PMA was carried out using an MWCNT paper (0.5 × 2 cm), an Ag/AgCl, and Pt wire as the working, reference, and counter electrodes, respectively^26^. CA and gas chromatography (GC) analyses were carried out using an H-Cell with two compartments separated by a Nafion membrane to prevent reduction of re-oxidized PMA on the cathode. The cathodic and anodic compartments were filled, respectively, with 0.5 M H_2_SO_4_ and 0.25 M PMA, which was pre-reduced by biomass in 0.5 M H_2_SO_4_. To prevent competition between reductions of protons and PMAs on a cathode, a Nafion membrane separate the anodic and cathodic half cells. Potentials vs. Ag/AgCl were converted to those vs. RHE using the following equation: *E* (V vs. RHE) = *E* (V vs. Ag/AgCl) + 0.0592 × *pH* + 0.197. No iR-compensation was conducted throughout the experiments.

### Photoelectrochemical characterization

PEC analysis was carried out in a three-electrode configuration with a photocathode, Pt wire, and Ag/AgCl (1 M KCl) as the working, counter, and reference electrodes, respectively. Bias-free PEC cells were tested in a homemade two-compartment cell under the following condition: a perovskite photocathode in the cathodic compartment was filled with 0.5 M H_2_SO_4_, and an MWCNT paper in the anodic compartment was filled with 0.25 M PMA that was pre-reduced by biomass in 0.5 M H_2_SO_4_. A Nafion membrane separated the two compartments. A 300-W Xe lamp equipped with AM 1.5 G filter (100 mW cm^−2^) was used as a visible light source. Samples for GC analysis were collected from the headspace of a sealed H-cell using a gas-tight syringe and were analyzed with a GC-2010 Plus gas chromatograph (Shimadzu Co., Japan). The perovskite photocathode’s IPCE efficiency was determined by using a 300-W Xenon lamp equipped with a 20 nm bandwidth monochromator. For day and night experiment, PMA solution was collected after PEC reaction and re-oxidized with the addition of 50 mg of lignin at 60 °C for 4 h to obtain the initial percentage of reduced PMA.

## Supplementary information

Supplementary Information

Description of Additional Supplementary Files

Supplementary Movie 1

## Data Availability

The experimental data generated in this study are provided in the Supplementary Information/Source Data file. Source data are provided with this paper.

## Source data

Source Data

## References

[1] Turner JA (2004). Sustainable hydrogen production. Science.

[2] Lewis NS, Nocera DG (2006). Powering the planet: chemical challenges in solar energy utilization. Proc. Natl Acad. Sci. USA..

[3] Walter MG (2010). Solar water splitting cells. Chem. Rev..

[4] Koper MT (2013). Theory of multiple proton–electron transfer reactions and its implications for electrocatalysis. Chem. Sci..

[5] Jiao Y, Zheng Y, Jaroniec M, Qiao SZ (2015). Design of electrocatalysts for oxygen-and hydrogen-involving energy conversion reactions. Chem. Soc. Rev..

[6] McCrory CC (2015). Benchmarking hydrogen evolving reaction and oxygen evolving reaction electrocatalysts for solar water splitting devices. J. Am. Chem. Soc..

[7] An L (2021). Recent development of oxygen evolution electrocatalysts in acidic environment. Adv. Mater..

[8] Holade Y (2020). Recent advances in the electrooxidation of biomass-based organic molecules for energy, chemicals and hydrogen production. Catal. Sci. Technol..

[9] Chen Y (2014). Nanotechnology makes biomass electrolysis more energy efficient than water electrolysis. Nat. Commun..

[10] Luo H (2021). Progress and perspectives in photo‐and electrochemical‐oxidation of biomass for sustainable chemicals and hydrogen production. Adv. Energy Mater..

[11] Wang W (2019). Modulation of molecular spatial distribution and chemisorption with perforated nanosheets for ethanol electro‐oxidation. Adv. Mater..

[12] Hayashi E (2019). Effect of MnO_2_ crystal structure on aerobic oxidation of 5-hydroxymethylfurfural to 2,5-furandicarboxylic acid. J. Am. Chem. Soc..

[13] Cha HG, Choi K-S (2015). Combined biomass valorization and hydrogen production in a photoelectrochemical cell. Nat. Chem..

[14] Liu W-J (2020). Efficient electrochemical production of glucaric acid and H_2_ via glucose electrolysis. Nat. Commun..

[15] Park Y, McDonald KJ, Choi K-S (2013). Progress in bismuth vanadate photoanodes for use in solar water oxidation. Chem. Soc. Rev..

[16] Zhang G, Liu G, Wang L, Irvine JT (2016). Inorganic perovskite photocatalysts for solar energy utilization. Chem. Soc. Rev..

[17] Wang S (2016). Synergistic crystal facet engineering and structural control of WO_3_ films exhibiting unprecedented photoelectrochemical performance. Nano Energy.

[18] Kim JH, Hansora D, Sharma P, Jang J-W, Lee JS (2019). Toward practical solar hydrogen production–an artificial photosynthetic leaf-to-farm challenge. Chem. Soc. Rev..

[19] Kim TW, Choi K-S (2014). Nanoporous BiVO_4_ photoanodes with dual-layer oxygen evolution catalysts for solar water splitting. Science.

[20] Kim JH (2016). Hetero-type dual photoanodes for unbiased solar water splitting with extended light harvesting. Nat. Commun..

[21] Yang WS (2015). High-performance photovoltaic perovskite layers fabricated through intramolecular exchange. Science.

[22] Stranks SD (2013). Electron-hole diffusion lengths exceeding 1 micrometer in an organometal trihalide perovskite absorber. Science.

[23] Kim J-H (2021). Efficient and stable perovskite-based photocathode for photoelectrochemical hydrogen production. Adv. Funct. Mater..

[24] Bhattacharjee S (2022). Reforming of soluble biomass and plastic derived waste using a bias‐free Cu30Pd70| perovskite| Pt photoelectrochemical device. Adv. Funct. Mater..

[25] Liu W (2014). Solar-induced direct biomass-to-electricity hybrid fuel cell using polyoxometalates as photocatalyst and charge carrier. Nat. Commun..

[26] Ding Y, Du B, Zhao X, Zhu J, Liu D (2017). Phosphomolybdic acid and ferric iron as efficient electron mediators for coupling biomass pretreatment to produce bioethanol and electricity generation from wheat straw. Bioresour. Technol..

[27] Zu X (2020). Ferric-ferrous redox couple mediated low temperature symmetric flow fuel cell for direct conversion of biomass residues into electricity. J. Power Sources.

[28] Oh H (2020). Phosphomolybdic acid as a catalyst for oxidative valorization of biomass and its application as an alternative electron source. ACS Catal..

[29] Le Floch A, Jourdes M, Teissedre P-L (2015). Polysaccharides and lignin from oak wood used in cooperage: Composition, interest, assays: A review. Carbohydr. Res..

[30] Novaes E, Kirst M, Chiang V, Winter-Sederoff H, Sederoff R (2010). Lignin and biomass: a negative correlation for wood formation and lignin content in trees. Plant Physiol..

[31] Wang Y, Sun S, Li F, Cao X, Sun R (2018). Production of vanillin from lignin: the relationship between β-O-4 linkages and vanillin yield. Ind. Crops Prod..

[32] Lourenço A, Araújo S, Gominho J, Evtuguin D (2020). Cellulose structural changes during mild torrefaction of Eucalyptus wood. Polymers.

[33] Sun S (2014). The origin of high efficiency in low-temperature solution-processable bilayer organometal halide hybrid solar cells. Energy Environ. Sci..

[34] Li F, Liu M (2017). Recent efficient strategies for improving the moisture stability of perovskite solar. J. Mater. Chem. A.

[35] Di Girolamo D (2020). Ion migration‐induced amorphization and phase segregation as a degradation mechanism in planar perovskite solar cells. Adv. Energy Mater..

[36] Khenkin MV (2020). Consensus statement for stability assessment and reporting for perovskite photovoltaics based on ISOS procedures. Nat. Energy.

[37] Verlage E (2015). A monolithically integrated, intrinsically safe, 10% efficient, solar-driven water-splitting system based on active, stable earth-abundant electrocatalysts in conjunction with tandem III–V light absorbers protected by amorphous TiO_2_ films. Energy Environ. Sci..

[38] Wang Y, Schwartz J, Gim J, Hovden R, Mi Z (2019). Stable unassisted solar water splitting on semiconductor photocathodes protected by multifunctional GaN nanostructures. ACS Energy Lett..

[39] Chen, Z., Dinh, H. N. & Miller, E. Photoelectrochemical water splitting (Springer, 2013).

