# Supplementary material for: Bias-free solar hydrogen production at 19.8 mA cm−2 using perovskite photocathode and lignocellulosic biomass
Source: Nat Commun. 2022 Oct 3;13:5709. doi: 10.1038/s41467-022-33435-1 (PMC9529942; doi:10.1038/s41467-022-33435-1)
Supplement: Supplementary file 1 — Supplementary Information [file 41467_2022_33435_MOESM1_ESM.pdf]

Supplementary Information

Bias-free solar hydrogen production at 19.8 mA/cm^2 using perovskite photocathode and lignocellulosic biomass

Yuri Choi[1,2]†, Rashmi Mehrotra[1,2]†, Sang-Hak Lee[1,2]†, Trang Vu Thien Nguyen[2], Inhui Lee[1,2], Jiyeong Kim[1,2], Hwa-Young Yang[1,2], Hyeonmyeong Oh[1,2], Hyunwoo Kim[1,2], Jae-Won Lee[3,4], Yong Hwan Kim[2,5], Sung-Yeon Jang[1,2,5]*, Ji-Wook Jang[1,2,5,6]*, Jungki Ryu[1,2,5,6]*

[1]Department of Energy Engineering, Ulsan National Institute of Science and Technology (UNIST), Ulsan 44919, Republic of Korea.

[2]School of Energy and Chemical Engineering, UNIST, Ulsan 44919, Republic of Korea.

[3]Department of Wood Science and Engineering, College of Agriculture & Life Sciences, Chonnam National University, Gwangju 61186, Republic of Korea.

[4]Interdisciplinary Program in IT-Bio Convergence System, Chonnam National University, Gwangju 61186, Republic of Korea.

[5]Graduate School of Carbon Neutrality, UNIST, Ulsan 44919, Republic of Korea.

[6]Emergent Hydrogen Technology R&D Center, UNIST, Ulsan 44919, Republic of Korea.

†These authors contributed equally to this work.

*Corresponding authors. Email: jryu@unist.ac.kr (J.R.); jiwjang@unist.ac.kr (J.-W.J.); syjang@unist.ac.kr (S.-Y.J.)

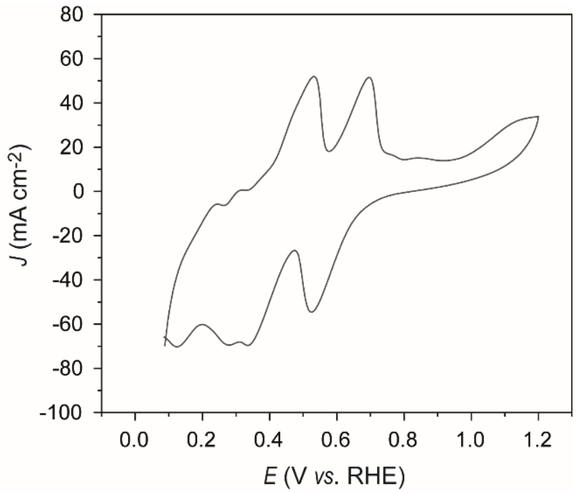

Supplementary Fig. 1 | Cyclic voltammogram of PMA showing its reversible redox activity. The electrolyte was 0.25 M PMA in 0.5 M H_2SO_4 and scan rate was 10 mV s^{-1}. CV analysis showed that PMA undergoes reversible redox behavior. The reversible redox behavior of PMA can also be found elsewhere[1, 2].

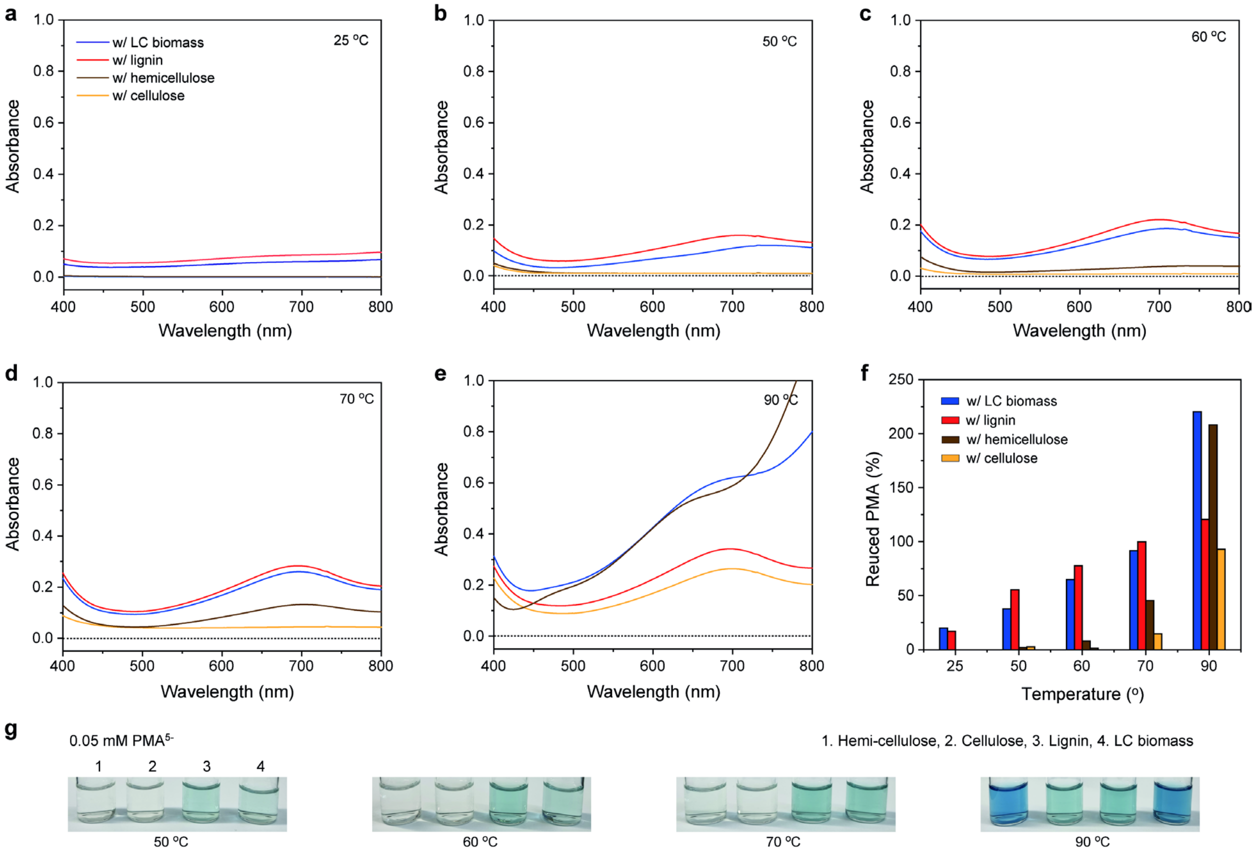

Supplementary Fig. 2 | Reduction of PMA^{3-} to PMA^{5-} via the oxidation of various biomass. UV/Vis absorbance spectra of PMA solutions that were incubated with LC biomass, lignin, hemicellulose, and cellulose oxidation at (a) 25 °C, (b) 50 °C, (c) 60 °C, (d) 70 °C, and (e) 90 °C for 8 h. (f) Effect of the type of biomass and incubation temperature on the reduction of PMA, which was calculated based on UV/Vis absorbance at 700 nm. Note that a degree of reduction higher than 100% implies that PMA underwent the reduction with more than two electrons and protons. (g) Photographs of the PMA solutions that were incubated with various biomass at different temperatures. The PMA solutions were diluted to 0.05 M to show clear differences.

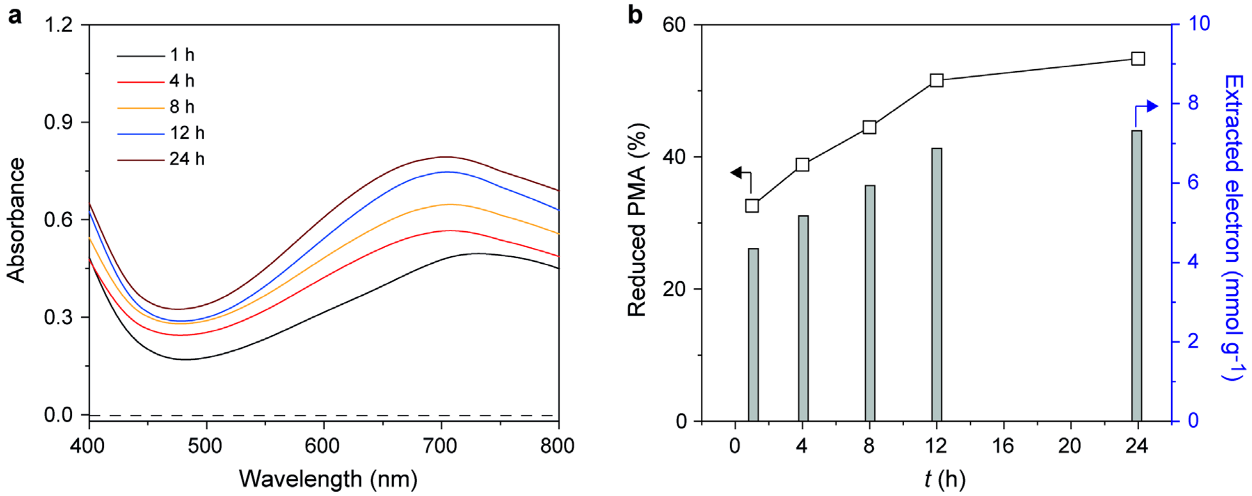

Supplementary Fig. 3 | Profile showing the reduction of PMA upon reaction with LC biomass. (a) UV/Vis absorbance spectra of PMA solutions that were pre-incubated with LC biomass oxidation. The mixed solutions of PMA (0.25 M) and biomass (37.5 mg mL^{-1}) were incubated at 60 °C for various reaction times. (b) Amounts of PMA reduced by and electrons extracted from LC biomass.

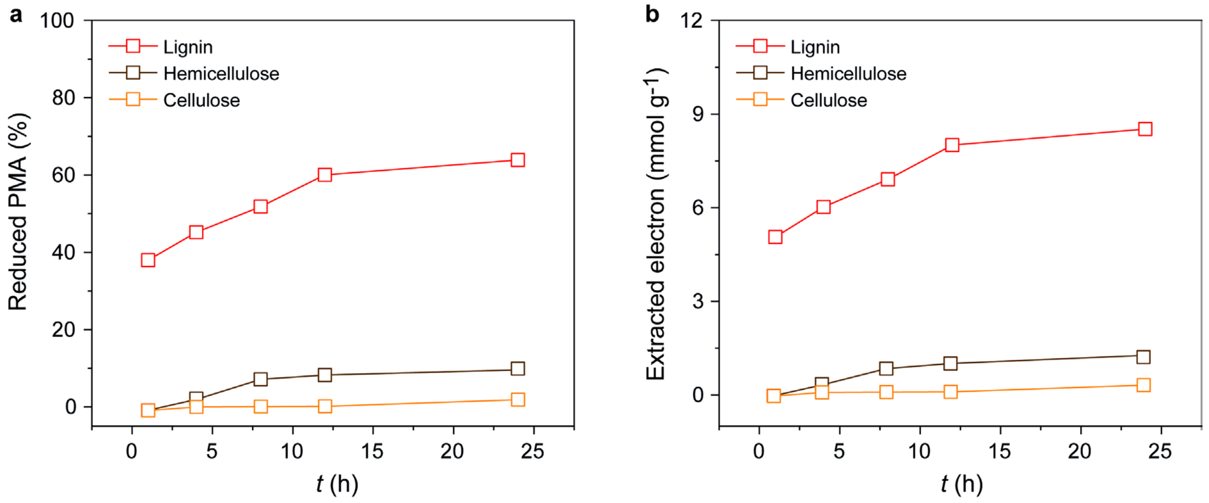

Supplementary Fig. 4 | Extraction of electrons from various biomass using PMA at 60 °C. (a) The degree of the PMA reduction (%) by lignin, hemicellulose, and cellulose oxidation at 60 °C, respectively. We used 375 mg of each biomass for the reaction with 0.25 M PMA (10 mL). (b) Amounts of electrons extracted per unit mass of lignin, hemicellulose, and cellulose oxidation.

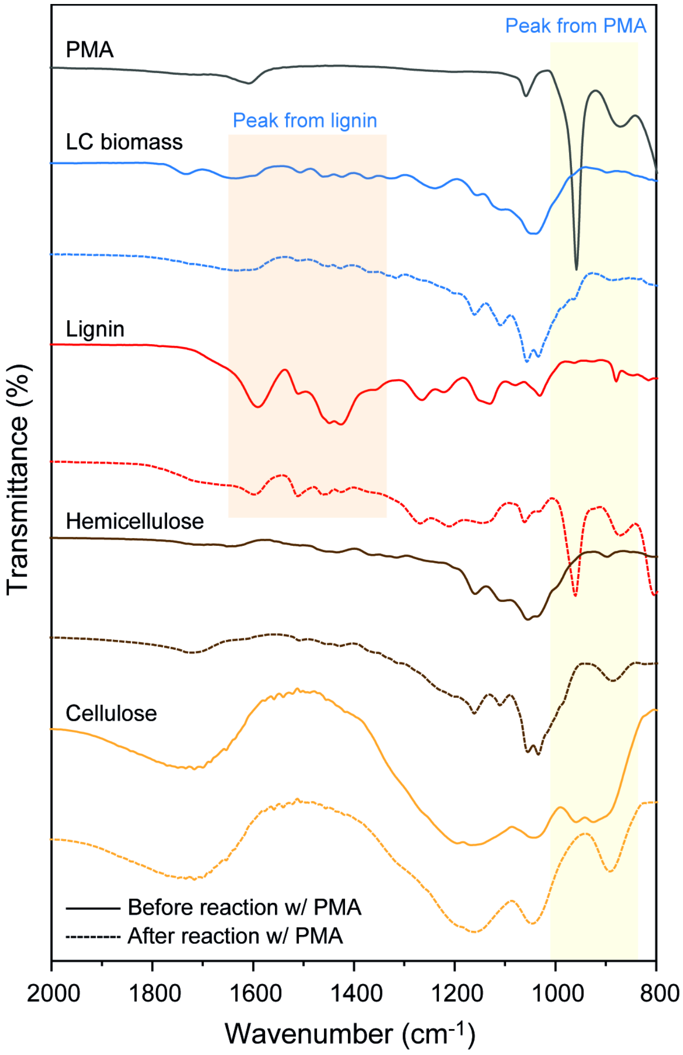

Supplementary Fig. 5 | Fourier-transform infrared (FT-IR) spectra of various biomass before and after oxidation by 0.25 M PMA at 60 °C for 8 h. After oxidation, the peaks at 1400–1800 cm^{-1} were substantially reduced in LC biomass and lignin due to the depolymerization of lignin. On the contrary, the peaks around 1100 cm^{-1}, corresponding to cellulose and hemicellulose, showed a negligible change, implying that these remained almost intact at 60 °C.

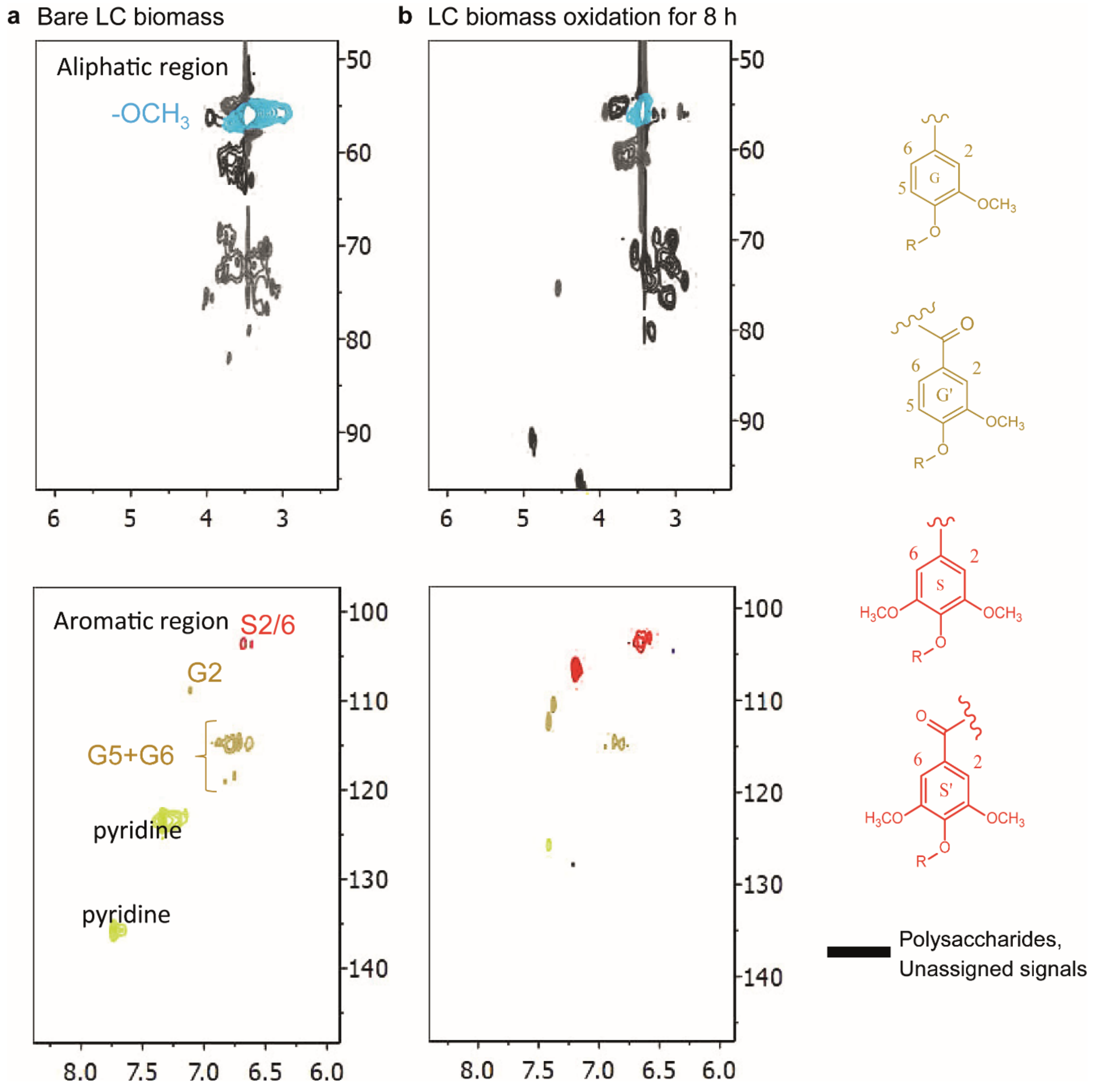

Supplementary Fig. 6 | Effect of PMA treatment on LC biomass structure. 2D NMR spectra of LC biomass (a) before and (b) after reaction with PMA at 60 °C for 8 h. After LC biomass oxidation at 60 °C for 8 h, the intensity of G peak was reduced, while other peaks remained unchanged. The G relates to lignin. This result supports that lignin in LC biomass was selectively depolymerized in the presence of PMA.

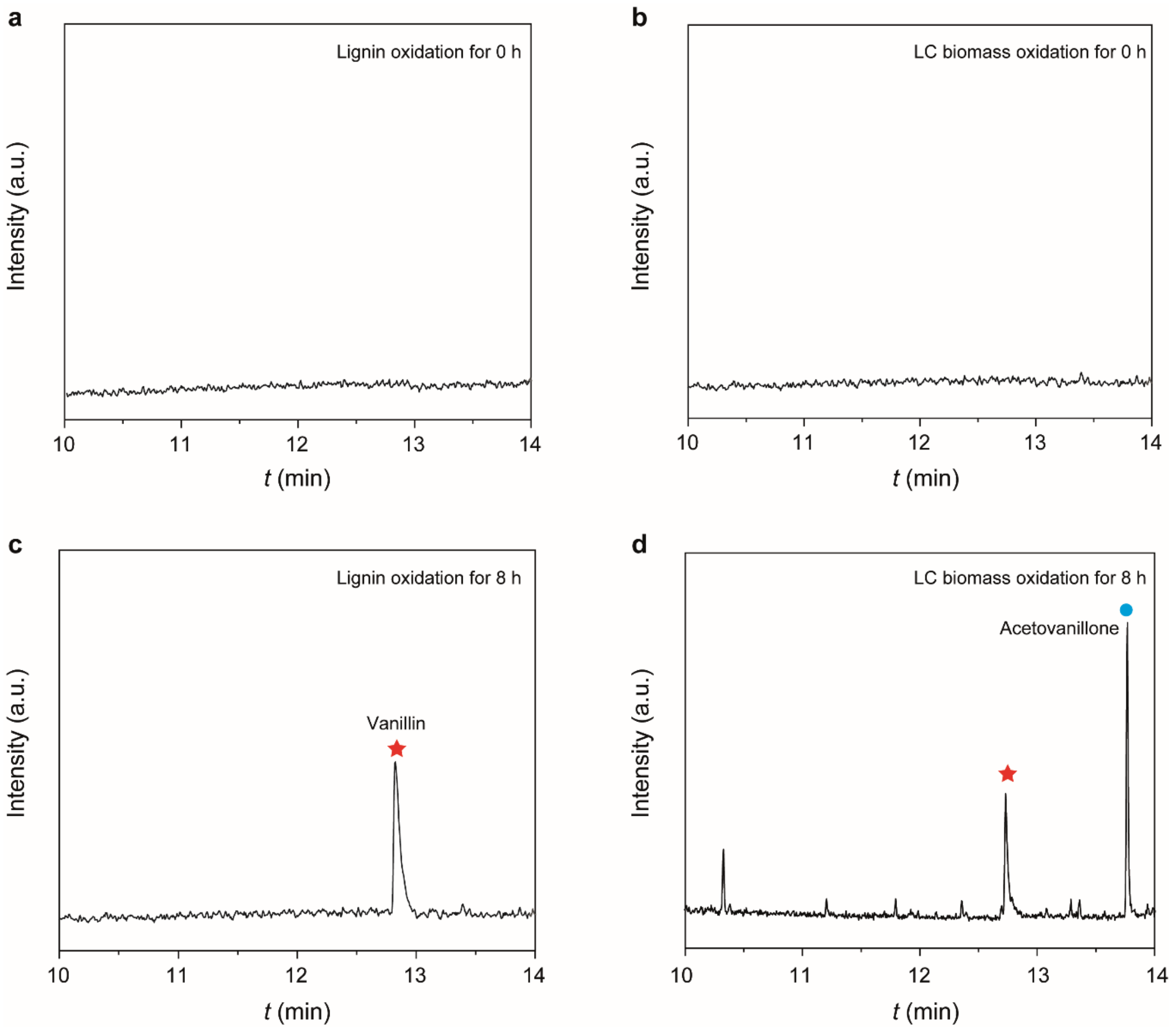

Supplementary Fig. 7 | Identification and quantification of aromatic compounds produced from the oxidation of lignin and LC biomass by GC-MS analysis. Soluble aromatic compounds from lignin and LC biomass were extracted using chloroform and then analyzed by GC-MS (a, b) before and (c, d) after the reaction with PMA at 60 °C for 8 h. Vanillin and acetovanillone were detected at 12.7 and 13.8 min, respectively. LC biomass and lignin produced 34.1 and 43.6 mg of vanillin per 1 g biomass, respectively, after reaction with PMA for 8 h. No vanillin was detected before the reaction. LC biomass additionally produced 44.3 mg of acetovanillone.

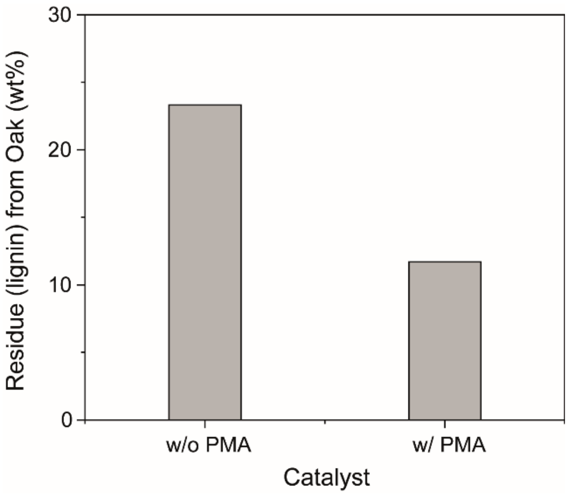

Supplementary Fig. 8 | Lignin contents in LC biomass from oak before and after reaction with PMA. Lignin content determined by the Klason method[3]. Pristine oak contained 23.7% of lignin, which was reduced to 11.7% after the reaction with PMA at 60 °C for 8 h.

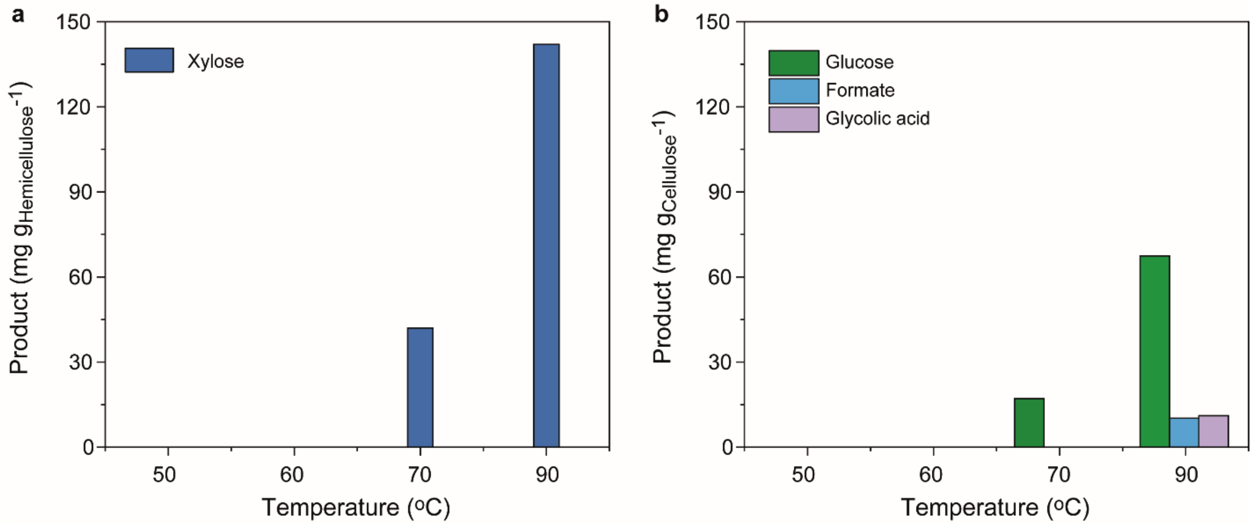

Supplementary Fig. 9 | LC Analysis of water-soluble compounds from hemicellulose and cellulose after reaction with PMA. Byproducts from (a) 375 mg of hemicellulose and (b) 375 mg of cellulose after reaction with 0.25 M PMA at different temperatures for 8 h. Hemicellulose and cellulose start substantially depolymerizing above 90 °C through the cleavage of C–OH, C–O–C, and C–C bonds.

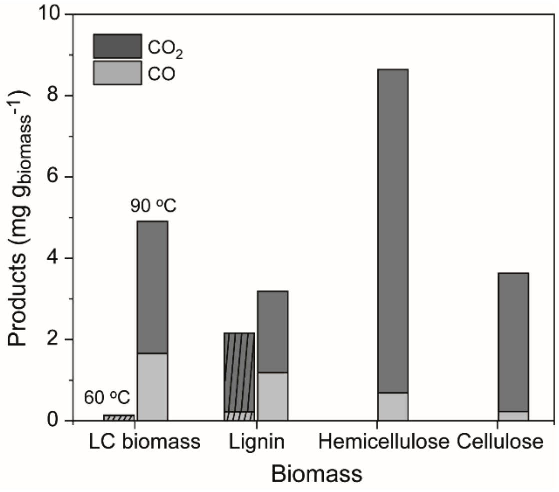

Supplementary Fig. 10 | GC Analysis of CO and CO_2 gas from LC biomass, lignin, hemicellulose, and cellulose after reaction with PMA. CO and CO_2 were produced during depolymerization of LC biomass, lignin, hemicellulose, and cellulose with PMA at 60 and 90 °C for 8 h. At 90 °C, the amounts of CO and CO_2 dramatically increased in hemicellulose and cellulose due to depolymerization. These results supported our hypothesis that hemicellulose and cellulose were not depolymerized at 60 °C.

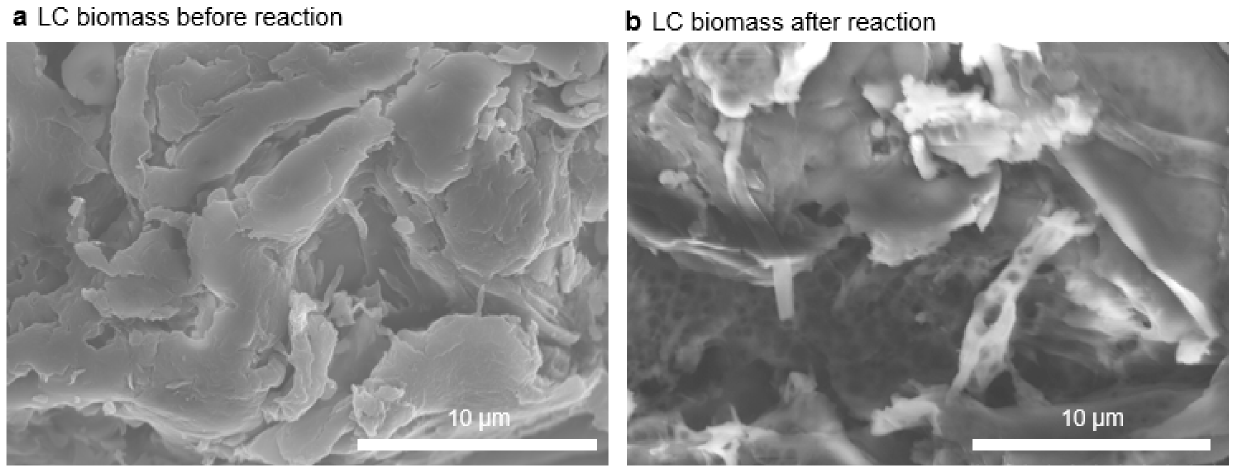

Supplementary Fig. 11 | Effect of treatment of LC biomass with PMA on its microstructure. Scanning electron microscopy (SEM) images of LC biomass (a) before and (b) after reaction with PMA at 60 °C for 8 h. After the reaction with PMA, a hierarchical porous structure was produced in LC biomass, suggesting that cellulosic components remained almost intact due to the selective depolymerization of lignin in LC biomass.

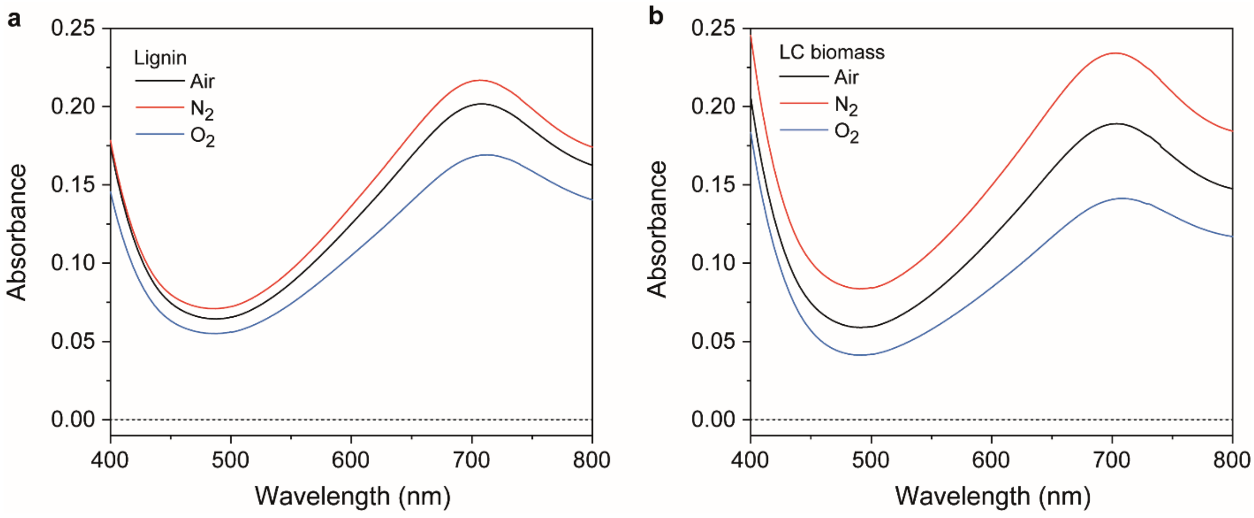

Supplementary Fig. 12 | Oxygen's effect on PMA reduction upon reaction with biomass at 60 °C for 8 h. Continuous O_2 purging led to a significant decrease in the amount of PMA reduced by (a) lignin (from 70.1% to 59.1%) and (b) LC biomass (from 64.8 to 45.2), compared with the air purging condition. The PMA solutions were diluted to 0.05 M to show clear differences.

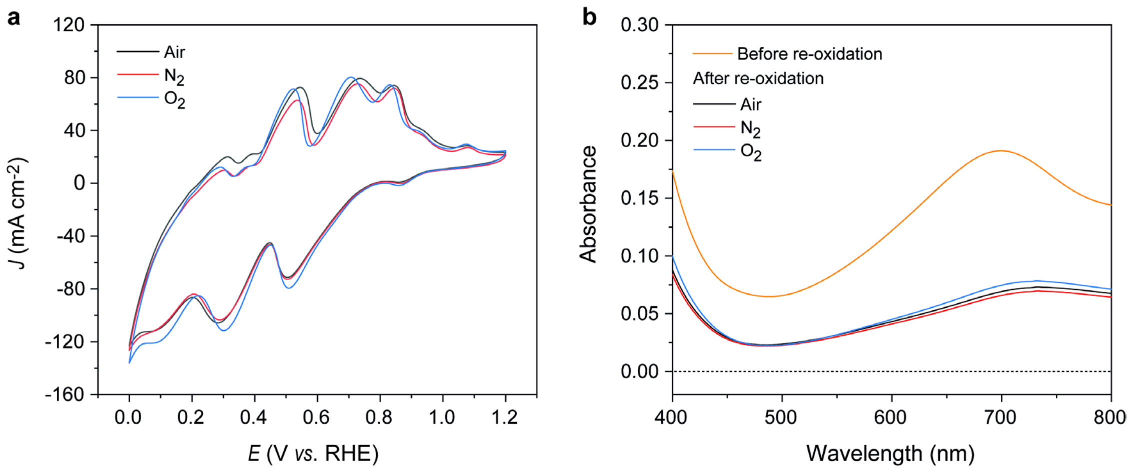

Supplementary Fig. 13 | Oxygen's effect on PMA re-oxidation upon reaction with biomass at 60 °C for 8 h. (a) CV graphs of reduced PMA solutions with a scan rate of 10 mV s^{-1} under air, N_2, and O_2 purging, respectively. (b) UV/Vis absorbance spectra of reduced PMA solutions after PMA re-oxidation for 12 h at 0.8 V *vs*. RHE under air, N_2, and O_2 purging, respectively. When we checked the effect of O_2 during PMA^{5-} re-oxidation, the absorbance at 700 nm was similar in UV/Vis spectra. This result indicated that PMA re-oxidation was not affected by O_2, and thus we performed (photo) electrochemical reaction under aerobic condition.

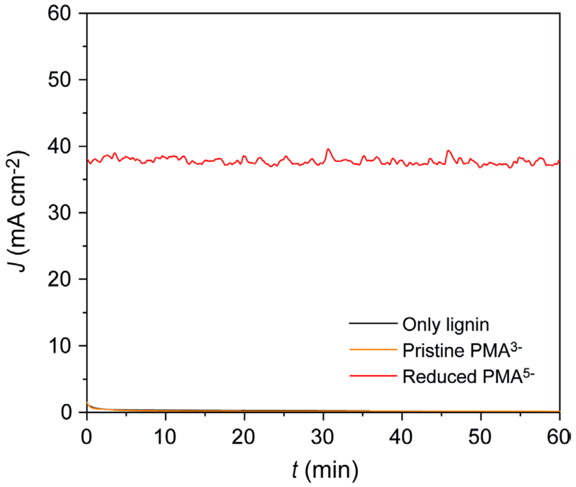

Supplementary Fig. 14 | Chronoamperograms of various anolyte solutions at 0.8 V *vs*. RHE. The anodic compartment of an H-cell was filled with only 375 mg of lignin, pristine 0.25 M PMA (3- form), or 0.25 M PMA reduced by lignin (5- form) at 60 °C for 8 h for CA, respectively.

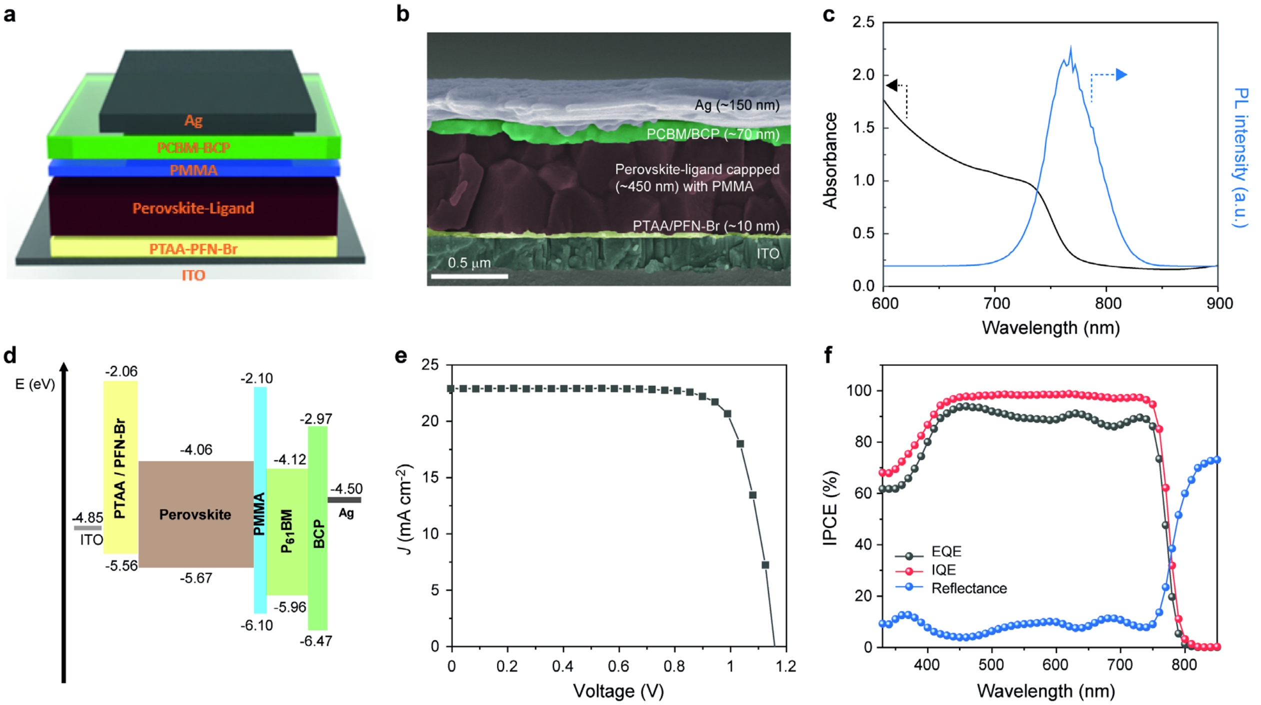

Supplementary Fig. 15 | Fabrication of perovskite solar cells. (a) Structure, (b) cross-sectional SEM image, (c) UV/Vis absorbance and photoluminescence spectra, (d) band diagram, (e) current density–voltage curve, and (f) IPCE spectra of the perovskite solar cell. To stabilize the perovskite film under the harsh acidic condition, we adopted bulk and top passivation of the perovskite layer, reducing non-radiative recombination and increasing the perovskite surface's hydrophobicity. For the bulk modification, a small amount of oleylamine ligand was added to the perovskite precursor. The ligand-capped crystal growth process improved crystal orientation and enhanced the grown perovskite's hydrophobicity, resulting in reduced non-radiative recombination and blocked water invasion of the perovskite layer[4]. For top passivation, the perovskite cell's surface was further treated with a low concentration of PMMA solution. This thin isolating layer can block the interfacial recombination between the holes in the perovskite and the electrons in the ETL and can suppress ion migration under continuous illumination. By using both techniques, the open-circuit potential reached 1.17 V, with a mean value of 1.14 ± 0.01 V.

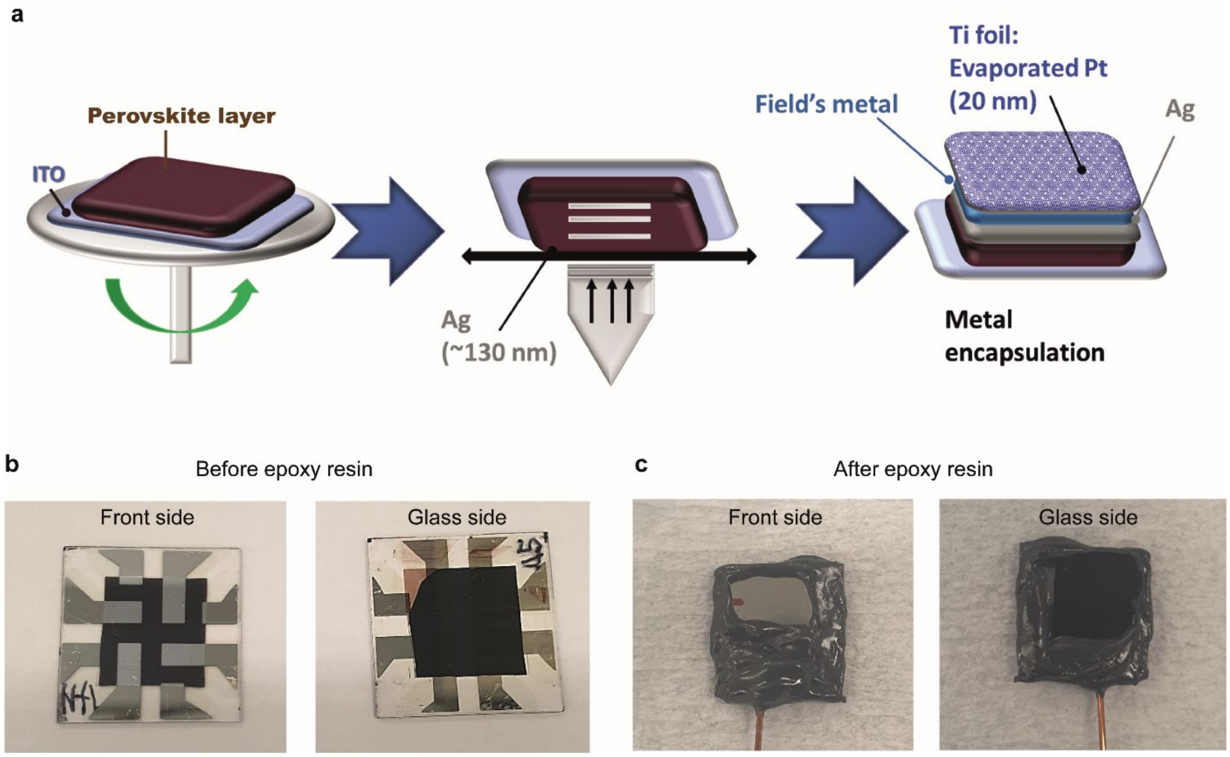

Supplementary Fig. 16 | Fabrication of Pt-Ti/FM/perovskite photocathodes for solar hydrogen production. (a) Step-by-step fabrication process for deposition of the Ag counter electrode on the perovskite solar cell by thermal evaporation, passivation with FM, decoration with the Pt–Ti foil as co-catalyst, and encapsulation with epoxy. Photographs of the Pt–Ti/FM/perovskite photocathodes (b) before and (c) after encapsulation with epoxy resin. Encapsulation at the edges was carried out precisely to prevent any air bubble trapping or electrolyte penetration. The photocathode was composed of indium-doped tin oxide (ITO)/PTAA:PFN-Br/$Cs_{0.05}(FA_{0.83}MA_{0.17})_{0.95}(PbI_{0.83}Br_{0.17})_3$ perovskite/PMMA-PCBM/BCP/Ag/FM/Ti-Pt layers.

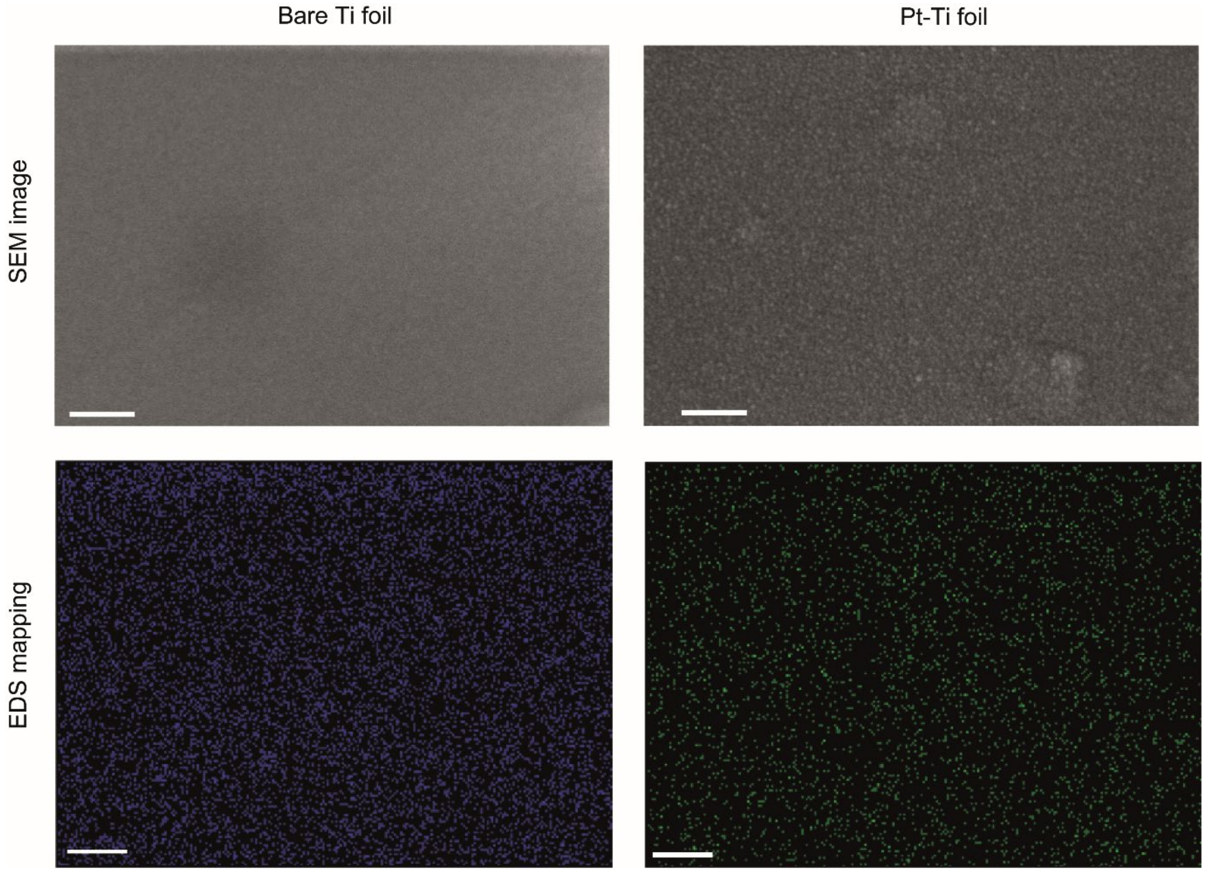

Supplementary Fig. 17 | Elemental mapping analysis of Pt-Ti foils. SEM and EDS elemental mapping analysis of Ti foil before and after deposition of Pt nanoparticles (20 nm). In EDS mapping images, blue and green dots represent Ti and Pt elements, respectively. All scale bars are 2 μm.

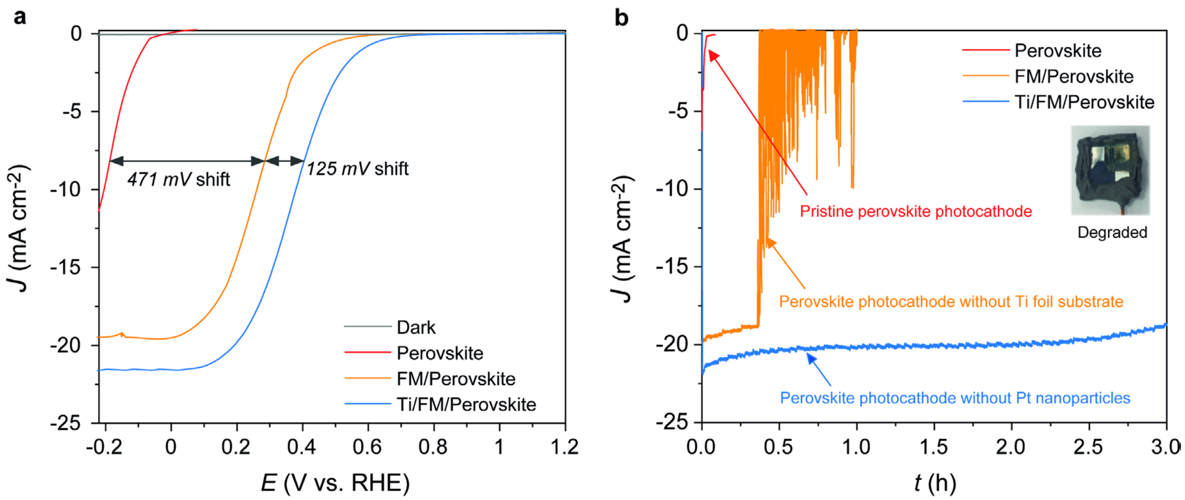

Supplementary Fig. 18 | Half-cell performance of perovskite photocathodes for solar hydrogen production. (a) Current density-voltage curves of various photocathodes: unencapsulated perovskite solar cell (perovskite), FM passivated perovskite (FM/Perovskite), and Ti foil encapsulated FM/perovskite (Ti/FM/perovskite). (b) Corresponding photocurrent profiles. The non-passivated perovskite solar cell films degrade within 5 min of exposure to an electrolyte. The Ti/FM/perovskite photocathode remained stable for 3 h, whereas the FM/Perovskite photocathode lost its photo-activity in 30 min due to the dissolution of FM. All the measurements were conducted in a 0.5 M H_2SO_4 solution (pH ~0.65) under ambient conditions in a simulated one-sun condition.

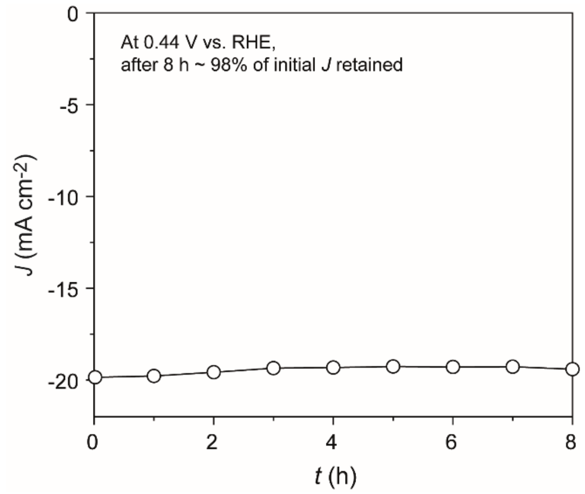

Supplementary Fig. 19 | Half-cell performance of the perovskite photocathode for solar hydrogen production in 0.5 M H_2SO_4. CA was measured using Pt-Ti/FM/perovskite as a working electrode at 0.44 V *vs*. RHE.

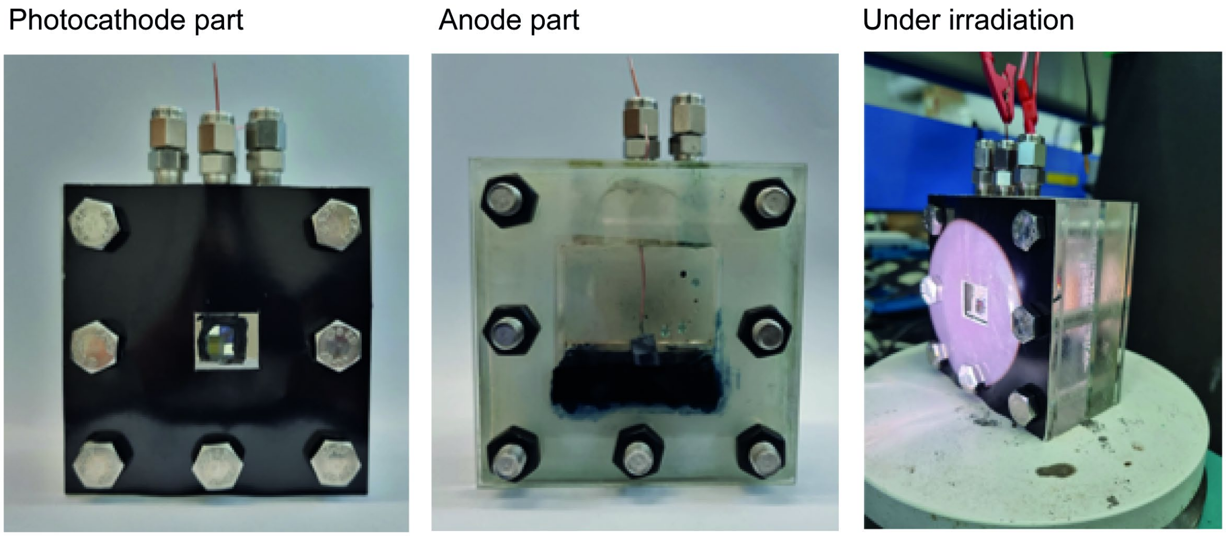

Supplementary Fig. 20 | Homemade two-compartment cell for unassisted solar hydrogen production. Bias free PEC cells were tested in a homemade two-compartment cell under the following conditions: a perovskite photocathode in the cathodic compartment was filled with 0.5 M H_2SO_4, and an MWCNT paper in the anodic compartment was filled with 0.25 M PMA— that was pre-reduced by biomass— in 0.5 M H_2SO_4. A nafion membrane separated the two compartments.

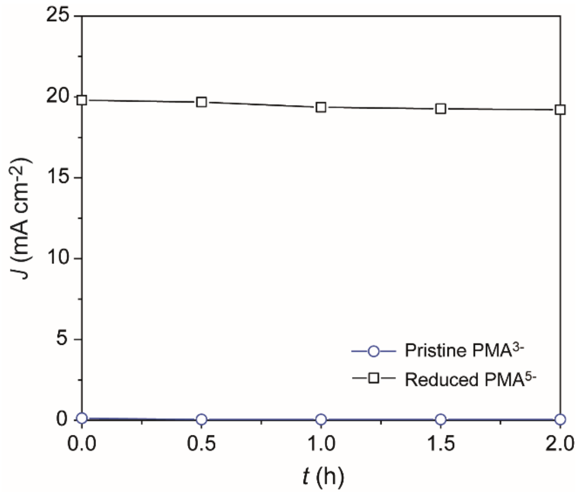

Supplementary Fig. 21 | Control experiment showing the role of reduced PMA as an alternative source of electrons and protons. No current flow was observed when using the pristine PMA^{3-} as an analyte in the two-electrode PEC test, whereas substantial current flow was observed when using the reduced PMA^{5-} without any applied bias.

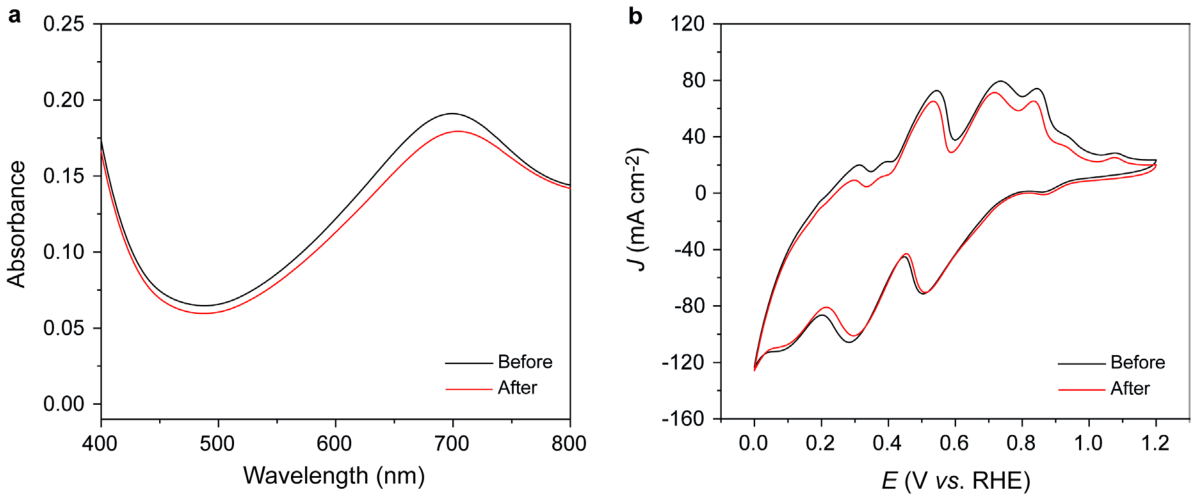

Supplementary Fig. 22 | Stability of PMA before and after long-term stability test for 20 h. (a) UV/Vis spectrum of the reduced PMA^{5-} before and after stability. The concentration of reduced PMA^{5-} by LC biomass was 0.05 M. The percentage of reduced PMA was reduced from 51.5% to 48.3%, which means 93.7% of reduced PMA was remained. (b) CV graphs of the reduced PMA^{5-} (0.25 M) before and after the stability test for 20 h. CV was measured at a scan rate of 10 mV s^{-1} of scan rate. These results demonstrated that the redox activity of PMA was maintained after the long-term stability test.

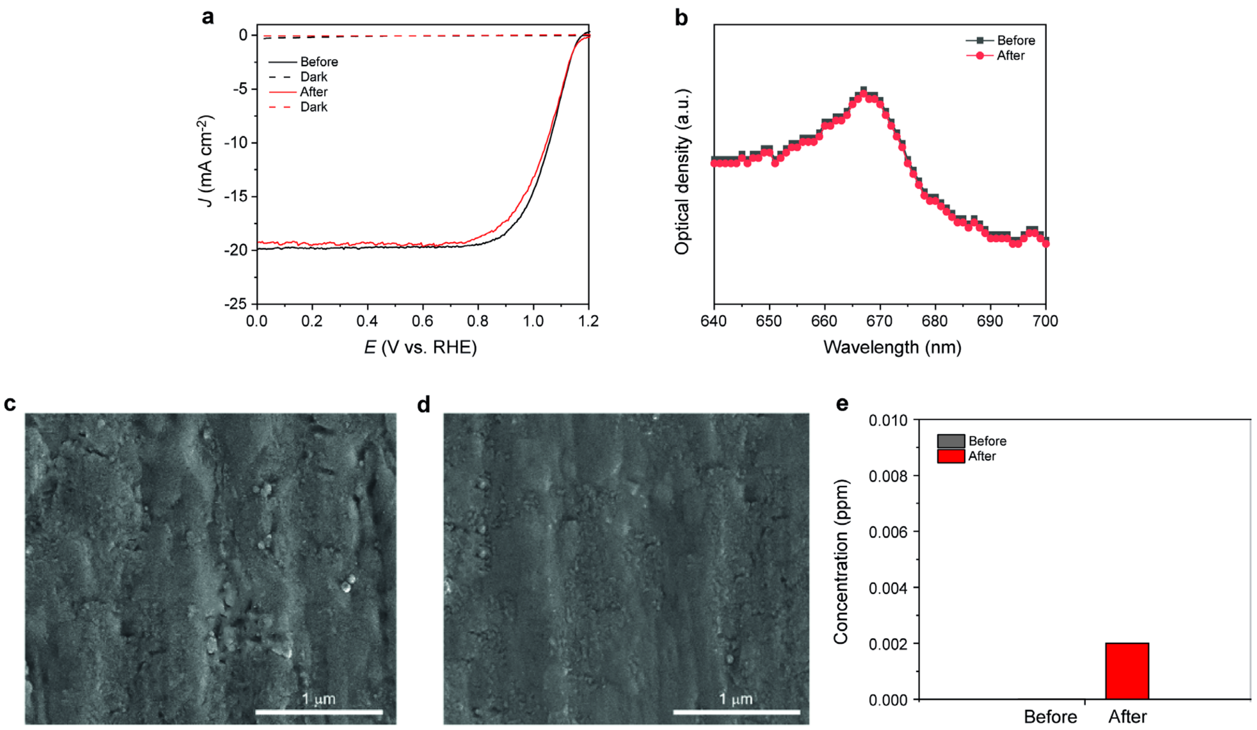

Supplementary Fig. 23 | Characterisation of the Pt-Ti/FM/Perovskite photocathode after 20 h of continuous illumination with simulated sunlight. (a) Polarization curve of the passivated Pt-Ti/FM/perovskite photocathode after stability test. (b) Optical density measurement of the passivated Pt-Ti/FM/Perovskite photocathode device before and after stability test conducted in two-electrode configuration. SEM images of the Pt-Ti before (c) and after (d) the long-term stability tests slight loss of the Pt particles from the reaction surface. (e) Inductively coupled plasma - optical emission spectrometry of the 0.5 M H_2SO_4 electrolyte used during measurement. Around 2 ppb of the Pt particles were found to be present in the electrolyte (10 mL) after the reaction was finished.

Supplementary Table 1 | Comparison between the performances of various PEC systems composed of single or dual photoelectrodes, and photovoltaic-assisted PEC systems.

No.	Anode	Cathode	Electrolyte	*J* (mA cm^{-2})	Product	STH (%)	Ref.
			Single photoelectrode				
0	CNT	Pt-Ti/FM/perovskite	0.5 M H_2SO_4	19.8	H_2, vanillin, acetovanillone	~12.3	This work
1	Pt	$In_{0.25}Ga_{0.75}N$	0.5 M H_2SO_4	2.7	H_2, O_2	~3.4	(5)
2	InGaP/GaAs tandem photoanode	Pt	0.5 M KOH	7.5	H_2, O_2	9	(6)
3	IrOx/3jn. III/V	Pt	3 M HCO_3	-	H_2, O_2	11.2	(7)
4	RuOx/3jn GaInP/GaInAs	Pt	3 M H_2SO_4	-	H_2, O_2	16.2	(8)
5	Co-Bi/3jn.Si	NiMoZn	1 M KBi (pH 9.2)	-	H_2, O_2	4.7	(9)
6	Ni/3jn. III/V	NiMo	1 M KOH	-	H_2, O_2	10.5	(10)
7	$GaInP_2$/GaAs/ Ge/GaN	Pt	0.1 M H_2SO_4	-	H_2, O_2	12.6	(11)
8	$Cu_{30}Pd_{70}$	Pt/perovskite	1 M KOH	~ 9	H_2, glycolic acid, gluconic acid, glyceric acid	-	(12)
			Dual photoelectrodes				
9	Pt/Si	Co_3O_4/Si	1.0 M KOH (pH = 14)	0.99	H_2, O_2	~0.61	(13)
10	CdS/TiO_2	CdSe/NiO	0.5 M Na_2SO_4 (pH=6.8)	0.19	H_2, O_2	~0.17	(14)
11	ZnO NRs	PEDOT:PSS/ P_3HT:PCBM/ Pt	0.25 M Na_2SO_4 (pH=6.8)	0.07	H_2, O_2	~0.12	(15)

12	$BiVO_4$	Cu_2O	0.2 M potassium borate (pH 9.0)	2.5	H_2, O_2	~3	(16)
13	NiFeOx/$BiVO_4$	Sb_2Se_3	H_2SO_4	1.2	H_2, O_2	~1.5	(17)
14	TiCo/$BiVO_4$	Pt/FM/Perovskite	0.1 M KBi, K_2SO_4 (pH 8.5)	0.49	H_2, O_2	0.49	(18)
15	$BiVO_4$/CoPi	Cu_2O	0.1 M Phosphate buffer	~0.41	H_2, O_2	~0.5	(19)
16	$BiVO_4$	Pt/CdS/CIGS	0.4 M K_2SO_4+0.4M K_2HPO_4 +0.4M KH_2PO_4	~3	H_2, O_2	3.7	(20)
17	$BiVO_4$	Pt/HfO_2/CdS/ Cu_2ZnSnS_4/Mo	0.2 M Sodium phosphate buffer (pH 6.5)	~0.85	H_2, O_2	1.046	(21)
18	Fe_2O_3	a-Si	0.5 M Phosphate (pH 11.8)	~0.7	H_2, O_2	~0.91	(22)
19	$BiVO_4$	ACGSe	0.1 M KH_2PO_4+NaOH (pH 7)	-	H_2, O_2	~0.67	(23)
20	$BiVO_4$	ZnSe-CIGS/ Ti-foil	1 M KPi (pH 7)	-	H_2, O_2	~1	(24)
Photovoltaic-assisted PEC systems							
21	IrO_2	Cu_2O/Perovskite solar cell	0.5 M Na_2SO_4+ 0.1 M phosphate (pH 5.0)	~2	H_2, O_2	~2.46	(25)
22	Ir and Ru coated Ti plate	Perovskite-Si dual-absorber tandem PEC cell	1.0 M H_2SO_4	11.3	-	~17.6	(26)
23	$BiVO_4$ + Solar cell	Pt	0.5 M KH_2PO_4 (pH = 7.3)	1.79	H_2, O_2	~2.21	(27)
24	TiO_2@$BiVO_4$/ Perovskite solar cell	Pt	0.1 M PBS (pH 7)	~1	H_2, O_2	~1.23	(28)
25	$CdIn_2S_4$/In_2S_3	Pt	0.5 M Na_2SO_4 (pH=6.8)	2.68	-	~3.3	(29)

26	$BiVO_4$‖Fe_2O_3-2p c-Si	Pt	1 M KCi (pH 9.2)	6.26	O_2	~7.7	(30)
27	WO_3/DSSC	Pt	1 M $HClO_4$ (pH 0)	-	-	~3.1	(31)
28	$BiVO_4$-2jn. Si	Pt	0.1 M Kbi (pH 7.3)	-	-	~4.9	(32)
29	(W, Mo)-doped $BiVO_4$/WO_3	Pt	0.5 M Na_2SO_4 (pH=6.8)	5.75	H_2	~7.1	(33)

Supplementary Table 2 | Comparison between the performances of biomass-PEC systems.

No.	Electrode	Electrolyte	Substrate	*t* (h)	H_2 (µmol cm^{-2} h^{-1})	Product	Ref.
Unbiased system							
0	CNT/Pt-Ti/FM/perovskite (single light absorber)	0.5 M H_2SO_4	LC biomass	10	512	vanillin, acetovanillone	This work
1	WO_3/CdTe/WC Tandem (dual light absorber)	0.33 M H_2SO_4	Glucose	-	-	CO_2	(34)
2	$Cu_{30}Pd_{70}$/ Pt/perovskite/ (single light absorber)	1 M KOH	Glucose	10	141.5	Gluconic acid	(12)
			Glycerol	10	108.3	Glyceric acid	
			Ethylene glycol	10	131.6	Glycolic acid	
			Cellulose	10	127.9	Gluconic acid	
Bias applied system							
3	C'@Cr-$SrTiO_3$/TiO_2 photoanode, Pt cathode (Applied bias – 0.3 V vs. SCE)	0.5 M KOH	Glucose	6	32.16	-	(35)
4	Pt/Bi_2WO_6 (Applied bias – 0.9 V vs. SHE)	0.1 M Na2SO4, phosphate buffer	Glucose	-	3.05	CO_2	(36)
5	CNT/Si (Applied bias – 0.8 V vs. RHE)	0.5 M H_2SO_4	Alkaline lignin	2	12	vanillin	(37)

Supplementary References

1. Symes, M. D. & Cronin, L. Decoupling hydrogen and oxygen evolution during electrolytic water splitting using an electron-coupled-proton buffer. *Nat. Chem.* **5**, 403-409 (2013).
2. Lu, L. & Xie, Y. Fabrication and supercapacitor behavior of phosphomolybdic acid/polyaniline/titanium nitride core–shell nanowire array. *New J. Chem.* **41**, 335-346 (2017).
3. Sluiter, A. *et al.*, *Determination of structural carbohydrates and lignin in biomass*. (Technical Report, NREL/TP-510-42618, National Renewable Energy Laboratory, 2008)
4. Zheng, X. *et al.*, Managing grains and interfaces via ligand anchoring enables 22.3%-efficiency inverted perovskite solar cells. *Nat. Energy* **5**, 131-140 (2020).
5. Wang, Y. *et al.* A single-junction cathodic approach for stable unassisted solar water splitting. *Joule* **3**, 2444-2456 (2019).
6. Varadhan, P., Fu, H. C., Kao, Y. C., Horng, R. H. & He, J. H. An efficient and stable photoelectrochemical system with 9% solar-to-hydrogen conversion efficiency via InGaP/GaAs double junction. *Nat. Commun.* **10**, 5282 (2019).
7. Okamoto, S., Deguchi, M. & Yotsuhashi, S. Modulated III–V triple-junction solar cell wireless device for efficient water splitting. *J. Phys. Chem. C* **121**, 1393-1398 (2017).
8. Young, J. L. *et al.* Direct solar-to-hydrogen conversion via inverted metamorphic multi-junction semiconductor architectures. *Nat. Energy* **2**, 17028 (2017).
9. Reece, S. Y. *et al.* Wireless solar water splitting using silicon-based semiconductors and earth-abundant catalysts. *Science* **334**, 645-648 (2011).
10. Verlage, E. *et al.* A monolithically integrated, intrinsically safe, 10% efficient, solar-driven water-splitting system based on active, stable earth-abundant electrocatalysts in conjunction with tandem III–V light absorbers protected by amorphous TiO_2 films. *Energy. Environ. Sci.* **8**, 3166-3172 (2015).
11. Wang, Y., Schwartz, J., Gim, J., Hovden, R. & Mi, Z. Stable unassisted solar water splitting on semiconductor photocathodes protected by multifunctional GaN nanostructures. *ACS Energy Lett.* **4**, 1541-1548 (2019).
12. Bhattacharjee, S. *et al.* Reforming of soluble biomass and plastic derived waste using a bias-free $Cu_{30}Pd_{70}$| perovskite| Pt photoelectrochemical device. *Adv. Funct. Mater.* **32**, 2109313 (2022).

13. Zhang, D. *et al.* Enabling unassisted solar water splitting by single-junction amorphous silicon photoelectrodes. *ACS Appl. Energy Mater.* **3**, 4629-4637 (2020).
14. Yang, H. B. *et al.* Stable quantum dot photoelectrolysis cell for unassisted visible light solar water splitting. *ACS Nano* **8**, 10403-10413 (2014).
15. Shao, D. *et al.* A spatially separated organic–inorganic hybrid photoelectrochemical cell for unassisted overall water splitting. *ACS Catal.* **7**, 5308-5315 (2017).
16. Pan, L. *et al.* Boosting the performance of Cu_2O photocathodes for unassisted solar water splitting devices. *Nat. Catal.* **1**, 412-420 (2018).
17. Yang, W. et al. Benchmark performance of low-cost Sb_2Se_3 photocathodes for unassisted solar overall water splitting. *Nat. Commun.* **11**, 861 (2020).
18. Andrei, V. *et al.* Scalable triple cation mixed halide perovskite-$BiVO_4$ tandems for bias-free water splitting. *Adv. Energy Mater.* **8**, 1801403 (2018).
19. Bornoz, P. *et al.* A bismuth vanadate–cuprous oxide tandem cell for overall solar water splitting. *J. Phys. Chem. C* **118**, 16959-16966 (2014).
20. Kobayashi, H. *et al.* Development of highly efficient $CuIn_{0.5}Ga_{0.5}Se_2$-based photocathode and application to overall solar driven water splitting. *Energy. Environ. Sci.* **11**, 3003-3009 (2018).
21. Huang, D. *et al.* Over 1% efficient unbiased stable solar water splitting based on a sprayed Cu_2ZnSnS_4 photocathode protected by a HfO_2 photocorrosion-resistant film. *ACS Energy Lett.* **3**, 1875-1881 (2018).
22. Jang, J. W. *et al.* Enabling unassisted solar water splitting by iron oxide and silicon. *Nat. Commun.* **6**, 7447 (2015).
23. Kim, J. H. *et al.* Overall photoelectrochemical water splitting using tandem cell under simulated sunlight. *ChemSusChem* **9**, 61-66 (2016).
24. Higashi, T. *et al.* Overall water splitting by photoelectrochemical cells consisting of $(ZnSe)_{0.85}(CuIn_{0.7}Ga_{0.3}Se_2)_{0.15}$ photocathodes and $BiVO_4$ photoanodes. *Chem. Commun.* **53**, 11674-11677 (2017).
25. Dias, P. *et al.* Transparent cuprous oxide photocathode enabling a stacked tandem cell for unbiased water splitting. *Adv. Energy Mater.* **5**, 1501537 (2015).
26. Karuturi, S. K. *et al.* Over 17% efficiency stand-alone solar water splitting enabled by perovskite-silicon tandem absorbers. *Adv. Energy Mater.* **10**, 2000772 (2020).

27. Peng, Y., Govindaraju, G. V., Lee, D. K., Choi, K. S. & Andrew, T. L. Integrating a semitransparent, fullerene-free organic solar cell in tandem with a $BiVO_4$ photoanode for unassisted solar water splitting. *ACS Appl. Mater. Interfaces* **9**, 22449-22455 (2017).
28. Zhang, X. *et al.* A perovskite solar cell-TiO_2@$BiVO_4$ photoelectrochemical system for direct solar water splitting. *J. Mater. Chem. A* **3**, 21630-21636 (2015).
29. Meng, L. *et al.* Designing a transparent $CdIn_2S_4/In_2S_3$ bulk-heterojunction photoanode integrated with a perovskite solar cell for unbiased water splitting. *Adv. Mater.* **32**, e2002893 (2020).
30. Kim, J. H. *et al.* Hetero-type dual photoanodes for unbiased solar water splitting with extended light harvesting. *Nat. Commun.* **7**, 13380 (2016).
31. Brillet, J. *et al.* Highly efficient water splitting by a dual-absorber tandem cell. *Nat. Photonics* **6**, 824-828 (2012).
32. Abdi, F. F. *et al.* Efficient solar water splitting by enhanced charge separation in a bismuth vanadate-silicon tandem photoelectrode. *Nat. Commun.* **4**, 2195 (2013).
33. Shi, X. *et al.* Unassisted photoelectrochemical water splitting exceeding 7% solar-to-hydrogen conversion efficiency using photon recycling. *Nat. Commun.* **7**, 11943 (2016).
34. Esposito, D. V. *et al.* Photoelectrochemical reforming of glucose for hydrogen production using a WO_3-based tandem cell device. *Energy Environ. Sci.* **5**, 9091-9099 (2012).
35. Zhang, Y. *et al.* Glucose oxidation over ultrathin carbon-coated perovskite modified TiO_2 nanotube photonic crystals with high-efficiency electron generation and transfer for photoelectrocatalytic hydrogen production. *Green Chem.* **18**, 2424-2434 (2016).
36. Madriz, L. *et al.* Photocatalysis and photoelectrochemical glucose oxidation on Bi_2WO_6: Conditions for the concomitant H_2 production. Renew. Energy **152**, 974-983 (2020).
37. Oh, H. *et al.* Phosphomolybdic acid as a catalyst for oxidative valorization of biomass and its application as an alternative electron source. *ACS Catal.* **10**, 2060-2068 (2020).
