# Supplementary material for: Bias-free solar hydrogen production at 19.8 mA cm−2 using perovskite photocathode and lignocellulosic biomass
Source: Nat Commun. 2022 Oct 3;13:5709. doi: 10.1038/s41467-022-33435-1 (PMC9529942; doi:10.1038/s41467-022-33435-1)
Supplement: Supplementary file 2 — Description of Additional Supplementary Files [file 41467_2022_33435_MOESM2_ESM.pdf]

File Name: Supplementary Movie 1
Description: **Bias-free solar hydrogen production using perovskite-based photocathode and lignocellulosic biomass**. Bias-free PEC cells were tested in a homemade two-compartment cell under the following condition: a perovskite photocathode in the cathodic compartment was filled with 0.5 M H_2SO_4, and an multi-walled carbon nanotube (MWCNT) paper electrode in the anodic compartment was filled with 0.25 M PMA that was pre-reduced by biomass in 0.5 M H_2SO_4.
